# Protocol for pooled FACS-based CRISPR knockout screening in human iPSC-derived microglia

**DOI:** 10.1016/j.xpro.2025.104111

**Published:** 2025-09-20

**Authors:** Sam J. Washer, Elena Navarro-Guerrero, Sally A. Cowley, Daniel V. Ebner, Andrew R. Bassett

**Affiliations:** 1 Wellcome Sanger Institute, Wellcome Genome Campus, Hinxton CB10 1SA, UK; 2 Open Targets, Wellcome Genome Campus, Hinxton CB10 1SA, UK; 3 James and Lillian Martin Centre for Stem Cell Research, Sir William Dunn School of Pathology, University of Oxford, South Parks Road, Oxford OX1 3RE, UK; 4 Target Discovery Institute, Centre for Medicines Discovery, Nuffield Department of Medicine, University of Oxford, Old Road Campus, Oxford OX3 7FZ, UK

**Keywords:** cell culture, CRISPR, high-throughput screening, sequencing, stem cells

## Abstract

Here, we present a protocol for CRISPR knockout screening in human induced pluripotent stem cell (hiPSC)-derived microglia (iMGL) using lentiviral delivery of CRISPR-Cas9 and co-transduction of VPX virus-like particles (VPX-VLPs). We first describe large-scale production of iMGL from hiPSCs, production of the lentiviral and VPX-VLP libraries, and titration. Next, we describe how to perform a pooled CRISPR screen for phagocytosis including the computational analysis pipeline of CRISPR screening data.

For complete details on the use and execution of this protocol, please refer to Perez-Alcantara et al.^1^

## Highlights

• Steps to perform CRISPR-Cas9 screening in human iPSC-derived microglia (hiPSC iMGL)
• High-throughput culture of iMGL from hiPSCs for CRISPR screening using bioreactors
• Production of VPX virus-like particles, CRISPR lentiviral libraries, and titration
• A phagocytosis CRISPR screen and step-by-step QC and analysis of CRISPR screen data

## Abstract (editor highlights)

Publisher’s note: Undertaking any experimental protocol requires adherence to local institutional guidelines for laboratory safety and ethics.

## Graphical abstract

**Figure:**
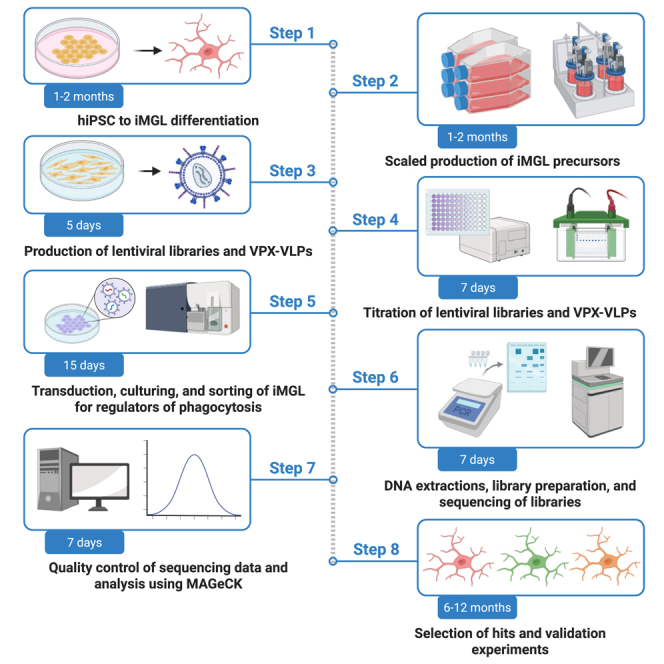

## Before you begin

Microglia are the resident macrophages of the brain responsible for a broad range of functions and have been associated with numerous neurodegenerative disorders such as Alzheimer’s Disease (AD), Parkinson’s disease, and Motor Neuron Disease.^2^^,^^3^^,^^4^^,^^5^^,^^6^ Therefore it is important to elucidate gene functions at scale. We have previously published a defined protocol for generating iMGL from various iPSC from different backgrounds through embryoid body generation and further maturation.^7^

**Figure 1:**
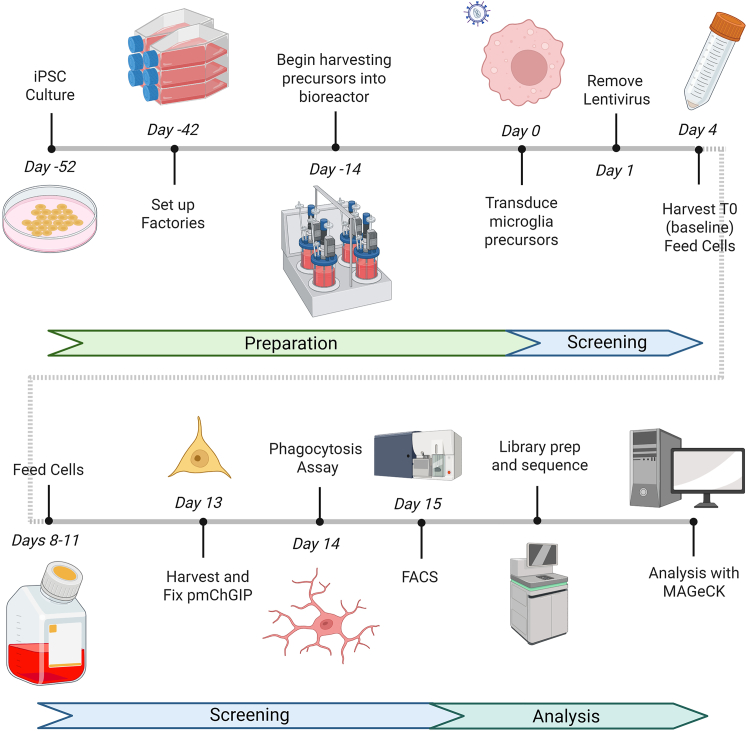
An overview of the pooled CRISPR screen for phagocytosis described in this manuscript

This protocol is an adaptation of a previous protocol designed for iPSC derived macrophages.^8^ iPSC are differentiated to microglia precursors through the formation of embryoid bodies, after several weeks microglia precursors are harvested and pooled together over multiple weeks to provide enough cells for screening. These microglia precursors are then transduced with a lentiviral library at the same time as a co-transducer (VPX-VLPs) while being plated in the final microglia media. After 14 days of differentiation to microglia, cells are ready for the user assay. The microglia can then be sorted, either live or fixed, into high and low bins before extracting DNA and amplicon sequencing for the guide sequences. Abundances of the guide sequences can then be calculated to examine the effect of genetic knockout on the phenotype in question. An overview of the protocol is provided in (Figure 1).

The library used in this example screen was a pooled viral vector comprised of 83 single guides (sgRNA) targeting 25 genes, including intergenic controls. The vector is a single all in one sgRNA CRISPR puromycin backbone henceforth known as all-in-one CRISPR sgRNA v3. This vector is a modification of the lentiCRISPRv2 backbone containing an optimized sgRNA backbone.^9^^,^^10^ We have also validated this method using the dual guide system, a single all-in-one CRISPR v3 backbone which contains two guides targeting the same gene for improved editing efficiency, henceforth known as all-in-one CRISPR dgRNA v3^11^.

The success of a pooled FACS based CRISPR screen is determined by the coverage of the lentiviral library through the protocol. We recommend the coverage of a single lentiviral vector should be approximately 300–500 cells at the endpoint of the screen per sorted population with exact numbers depending on the strength of the phenotype.

This means per sgRNA would require approximately 40,000–60,000 cells at the start. A helpful table for calculating cell numbers required for a pooled CRISPR screen is provided in Table S1.

## Key resources table

**Table:** 

REAGENT or RESOURCE	SOURCE	IDENTIFIER
**Antibodies**		

SAMHD1 anti-mouse (use at 1:500)	Abcam	Ab67820
GAPDH anti-rabbit (use at 1:1,000)	CST	14C10
Anti-rabbit IgG, HRP-linked antibody (use at 1:2,500)	CST	7074S
Rabbit anti-mouse IgG (light chain specific) (D3V2A) mAb (HRP conjugate) (use at 1:2,500)	CST	58802S

**Recombinant DNA**		

pSIV-D3psi/delta env/delta Vif/delta Vpr	Addgene	132928
psPAX2	Addgene	12260
pCMV-VSV-G	Addgene	8454
All in one V3 lentiviral library	In-house	Burgold et al.^11^

**Chemicals, peptides, and recombinant proteins**		

Essential E8	Gibco	A1517001
X-VIVO 15	Lonza	BE02-60F
ADMEM/F12	Life-Tech	12634-10
OptiMEM	Invitrogen	51985026
DMEM/F12	Gibco	31331093
GlutaMAX	Life-Tech	35050-061
DPBS	Thermo Fisher Scientific	14190094
Distilled water	Thermo Fisher Scientific	15230089
Y-27632	STEMCELL Technologies	72305
FBS	Merck	F9665
TrypLE Express	Thermo Fisher Scientific	12604021
Trypan blue stain (0.4%)	Gibco	15250-061
UltraPure 0.5 M EDTA, pH 8.0	Invitrogen	15575020
Vitronectin (VTN-N) recombinant human protein, truncated	Gibco	A14700
SCF	PeproTech	300-07-50UG
VEGF	PeproTech	100-20A-50UG
BMP-4	PeproTech	120-05ET
IL-3	Cell Guidance Systems	GFH80
β-mercaptoethanol	Gibco	31350-010
M-CSF	PeproTech	300-25-1MG
GM-CSF	PeproTech	300-03-50UG
IL-34	PeproTech	200-34
TGF-β1	PeproTech	100-21C
BSA	Merck	A9418-50G
1 M HCl	Sigma	H9892
Gelatin	Sigma	G1393
Lipofectamine-LTX	Invitrogen	15338100
Polybrene	Sigma	H9268
Puromycin	Sigma	P8833-25MG
Resazurin	Sigma	R7017
RIPA	Thermo Fisher Scientific	89900
cOmplete EDTA free protease inhibitors	Merck	11836170001
16% PFA	Thermo Fisher Scientific	043368.9M
Tris base	Merck	252859-500G
SDS	Merck	L3771-100G
Glycine	Merck	G7126-1KG
Methanol	Merck	179957-1L
Tween 20	Merck	
Glycogen solution	QIAGEN	158930
Proteinase K	QIAGEN	158146
RNase A	QIAGEN	158924
Isopropanol	VWR	20904.320
NEBNext Ultra 5 Q5 master mix	NEB	M0544X
Nuclease-free water	Applied Bioscience	AM9937
Sequencing primers	IDT	This paper
Gel loading dye, purple (6×), no SDS	NEB	B7025S
Sodium acetate	Invitrogen	AM9740
Qubit 1× dsDNA high sensitivity	Invitrogen	Q33230

**Other**		

Greiner cell culture multiwell plate, 6 well	Greiner	657160
Greiner cell culture multiwell plate, 12 well	Greiner	665180
Greiner cell culture flask, 550 mL 175 cm^2^	Greiner	660175
Corning cell culture flask, 175 cm^2^	Corning	431080
Low-attachment U-bottom 96-well plate	Corning	7007
15cm^2^ Nunc EasYDish dishes	Thermo Fisher Scientific	150468
Corning 1 L disposable spinner flask, vent cap, sterile	Corning	3580
Cimarec Biosystem slow-speed stirrer for cell culture	Thermo Fisher Scientific	50119114
Cimarec Biosystem 40B controller	Thermo Fisher Scientific	50118918
0.45 μm vacuum filters low bind SFCA	VWR	734-5068
EASYstrainer 40 μM for 50 mL tubes	Greiner	542040
BCA assay kit	Thermo Fisher Scientific	23225
Puregene cell kit	QIAGEN	158043
QIAquick gel extraction kit	QIAGEN	28704

**Experimental models: Cell lines**		

Human: HEK293T	ATCC	CRL-3216
Human: KOLF2.1J	JAX iPSC Catalog	JIPSC001000

**Software and algorithms**		

MAGeCK	Li et al.^12^	https://sourceforge.net/p/mageck/wiki/Home/
MAGeCK-flute	Wang et al.^13^	https://doi.org/10.18129/B9.bioc.MAGeCKFlute

## Materials and equipment

• Prepare a 10 mM stock of Y-27632 by adding 3.122 mL sterile water (Thermo Fisher Scientific, 15230089) to a 10 mg ampule of Stem Cell Technologies Y-27632 #72305. Vortex to mix and create 50 μL single use aliquots. Store at −20°C for 6 months.
• Prepare a 1:100 dilution of vitronectin by adding 10 μL vitronectin (Gibco, A14700) to 1 mL DBPS (no calcium, no magnesium).
• Prepare a 0.5 mM stock of EDTA DPBS by combining 50 μL UltraPure 0.5 M EDTA, pH 8.0 with 50 mL DPBS (no calcium, no magnesium), vortex to mix. Store at room temperature for 3 months.
• For growth factor reconstitution dissolve BSA (Merck A9418-50G) at a concentration of 100 g/L (10% w/v) in DPBS and sterilize by filtration through a 0.22 μm filter. Prepare 1 mL aliquots and store at −20°C for up to 1 year.
• Prepare a 10 μg/mL stock of SCF as follows. Centrifuge vial of SCF (PeproTech 300-07-50UG). Add 500 μL distilled water (ThermoFisher, 15230089) to make a 0.1 mg/mL stock. Prepare 5 mL 0.1% BSA/DPBS by adding 50 μL 10% BSA/DPBS in 4.95 mL DPBS. To the 0.1 mg/mL stock of SCF add 4.5 mL 0.1% BSA/DPBS and mix. Aliquot into 100 μL single use aliquots and store at −80°C for 12 months.
• Prepare a 25 μg/mL stock of VEGF. Prepare a 0.1% BSA/DPBS solution by adding 50 μL 10% BSA to 4.95 mL DPBS. Add 2 mL 0.1% BSA/DPBS to the 50 μg ampule of VEGF to make a 25 μg/mL stock. Create 100 μL single use aliquots and store at −80°C for 12 months.
• Prepare 25 μg/mL stock of BMP-4 as follows. Prepare a 5 mM HCl solution by adding 50 μL 1 M HCl (Sigma H9892) to 9.95 mL dH_2_O. To 4.95 mL 5 mM HCl add 50 μL 10% BSA/DPBS to make a 0.1% BSA 5 mM HCl solution. Add 200 μL of 0.1% BSA 5 mM HCl solution to a 50 μg ampule of BMP-4 to make a 250 μg/mL stock of BMP-4. Prepare a 0.1% BSA/DPBS solution by adding 50 μL 10% BSA/DPBS to 4.95 mL DPBS. Add 1.8 mL of 0.1% BSA/DPBS to the 200 μL 250 μg/mL stock of BMP-4 to get a final stock concentration of 25 μg/mL. Aliquot into 50 μL single use aliquots and store at −80°C for 3 months.
• Prepare 50 μg/mL IL-3 as follows. Prepare a 0.1% BSA/DPBS solution by adding 50 μL 10% BSA/DPBS to 4.95 mL DPBS. Add 500 μL dH_2_O to a 100 μg vial of IL-3 to make a 200 μg/mL stock. Further dilute by adding 1.5 mL 0.1% BSA/DPBS to make a 50 μg/mL stock solution. Aliquot into 250 μL single use aliquots and store at −80°C for 3 months.
• Prepare a 100 μg/mL stock of M-CSF as follows. To 1 mg of M-CSF (Peprotech 300-25-1MG) add 2 mL of dH_2_O to make a 500 μg/mL stock. Prepare 10 mL of 0.1% BSA/DPBS by adding 100 μL 10% BSA to 9.9 mL DPBS. Add 2 mL of 500 μg/mL M-CSF to 8 mL 0.1% BSA/DPBS and mix. Store in 500 μL aliquots for use in Factory media, and 50 μL aliquots for use in ITMG microglia media. Store at −80°C for 12 months.
• Prepare a 100 μg/mL stock of IL-34 as follows. To 250 μg IL-34 (Peprotech 200-34-250UG) add 250 μL dH_2_O to make a 1 mg/mL stock. Prepare 5 mL of 0.1% BSA/DPBS by adding 50 μL 10% BSA to 4.95 mL DPBS. Add 250 μL 1 g/mL IL-34 to 2.25 mL 0.1% BSA/DPBS to create 2.5 mL of 100 μg/mL stock. Store in 50 μL or 100 μL single use aliquots at −80°C for 3 months.
• Prepare a 50 μg/mL stock of TGFβ1 as follows. To 100 μg TGFβ1(Peprotech 100-021-100UG) add 500 μL dH_2_O to make a 200 μg/mL stock. Prepare 5 mL of 0.1% BSA/DPBS by adding 50 μL 10% BSA to 4.95 mL DPBS. Add 500 μL 200 μg/mL TGFβ1 to 1.5 mL 0.1% BSA/DPBS to create 2 mL of 50 μg/mL stock. Store in 50 μL or 100 μL single use aliquots at −80°C for 12 months.
• Prepare a 10 μg/mL stock of GM-CSF as follows. To 50 μg GM-CSF (Peprotech 300-03-50UG) add 100 μL dH_2_O to make a 500 μg/mL stock. Prepare 5 mL of 0.1% BSA/DPBS by adding 50 μL 10% BSA to 4.95 mL DPBS. Add 100 μL 500 μg/mL GM-CSF to 4.9 mL 0.1% BSA/DPBS to create 5 mL of 10 μg/mL stock. Store in 50 μL or 100 μL single use aliquots at −80°C for 12 months.
• Prepare 0.1% Gelatin (Sigma, G1393-100 mL) by diluting 26 mL of 2% gelatin stock in 500 mL of sterile water (Gibco). Warm to 37°C before use, store at 4°C for 6 months.
• For lentiviral resuspension dissolve BSA (Merck A9418-50G) at a concentration of 15 g/L (1.5% w/v) in 1× PBS and sterilize by filtration through a 0.22 μm filter. Store at 4°C for up to 1 week.
• Dissolve polybrene (Sigma, H9268) at a concentration of 10 mg/mL in 1× water and sterilize by filtration through a 0.22 μm filter. Store in single use aliquots at −20°C
• Dissolve puromycin (Sigma, P8833-25MG) in sterile 1× PBS at 1 mg/mL and store at −20°C
• Dissolve resazurin sodium salt (Sigma, R7017) at 10 mg/mL with sterile 1× PBS. Aliquot and store at −20°C.
• Prepare 4% PFA by diluting 10 mL of 16% PFA (ThermoFisher, 043368.9 M) in 30 mL DPBS (total volume 40 mL). Store at room temperature for 6 months.
• 10× Running buffer for western blot, pH to 8.3.

**Table:** 10× western blot running buffer

Reagent	Final concentration	Amount
Tris-Base	250 mM	30.29 g
Glycine	1.92 M	144.13 g
SDS	1% w/v	10 g
ddH_2_O	1×	1 L

Store at 15°C–25°C for 6 months.

• To make 1× western blot running buffer dilute 100 mL of 10× western blot running buffer with 900 mL water (final conc. 25 mM Tris-Base, 192 mM Glycine, 0.1% SDS). Store at 15°C–25°C for 6 months.
• 10× Transfer buffer for western blot.

**Table:** 10× western blot transfer buffer

Reagent	Final concentration	Amount
Tris-Base	250 mM	30.29 g
Glycine	1.92 M	144.13 g
SDS	0.5% w/v	5 g
ddH_2_O	1×	1 L

Store at 4°C for 6 months.

• 1× Transfer buffer for western blot.

**Table:** 1× western blot transfer buffer

Reagent	Final concentration	Amount
10× Transfer Buffer	1×	100 mL
Methanol	20% v/v	200 mL
ddH_2_O	1×	700 mL

Store at 4°C for 1 month.

• Phosphate buffered saline with tween wash buffer (PBST).

**Table:** 1× PBST

Reagent	Final concentration	Amount
10× PBS	1×	100 mL
TWEEN-20	0.01% v/v	1 mL
ddH_2_O	1×	1 L

Store at 15°C–25°C for 6 months.

## Step-by-step method details

### Culturing of hiPSCs and generation of iMGL precursors

**Timing: 1–2 months**

This section outlines the successful generation of iMGL precursors from hiPSC. Firstly, the initial thawing and recovery of hiPSC, then their differentiation into mesoderm embryoid bodies (EB) through low-attachment U bottom 96 well-plates, and finally the transfer of the EB into T175 flasks, henceforth known as “factories”, and the successful maintenance of these cells.

**CRITICAL:** The protocol requires significant incubator space for the generation of enough precursors for screening. Please ensure sufficient tissue culture space for the number of flasks required. A calculation sheet is provided in the Table S1 to calculate usage of cells.

***Note:*** We recommend undertaking CRISPR screens with the KOLF2.1J iPSC line from the Jackson laboratory.^14^ This line has been extensively characterized and is the recommended baseline iPSC model in order to produce reproducible results across institutes. However, this protocol can be utilized by alternative iPSC lines.

1. Thaw one vial of KOLF2.1J iPSC.
  a. Coat 6 well plate with 1 mL per well 1:100 dilution of vitronectin (made in DPBS), leave to coat for 1 h at room temperature.
  b. Warm aliquot of complete E8 media to room temperature.
  c. Prepare an aliquot of complete E8 containing 10 μM Y-27632 ROCKi.
  d. Thaw iPSC at 37°C for 5 min.
  e. Transfer thawed iPSC using a P1000 to a 15 mL tube containing 13 mL DPBS.
  f. Centrifuge cells at 400 g for 5 min at room temperature.
  g. Remove supernatant and resuspend the pellet in 1 mL complete E8 containing ROCKi from step c.
  h. Remove vitronectin from 6 well plates using an aspirator.
  i. Add 1.2 mL of E8 containing ROCKi from Step c to one coated well, and 1.8 mL to another.
  j. Add 800 μL cell suspension from Step i to the well containing 1.2 mL E8 with ROCKi and the remaining 200 uL cell suspension to the well containing 1.8 mL E8 with ROCKi.
  k. Agitate in a cross-hatch method and incubate cells at 37°C 5% CO_2_.
2. Check on the cells 24 h post thaw.

***Note:*** They should have formed well defined colonies containing a monoculture of cell morphologies, the inclusion of ROCKi will make the cells appear spiny. Following removal they will become rounded in morphology. Check both the 80% and 20% seeded wells

3. Remove 100% media and replace with 2 mL E8 without ROCKi.
4. Perform daily 100% media changes with 2 mL E8 until one well reaches 70%–80% confluency.
5. EDTA passaging of iPSC.
  a. Coat 6 well plate with 1 mL per well 1:100 dilution of vitronectin (made in DPBS), leave to coat for 1 h at room temperature.
  b. Remove media from cells.
  c. Wash with 1 mL DPBS using a P1000.
  d. Wash with 1 mL 0.5 mM EDTA DPBS using a P1000.
  e. Add 500 μL 0.5 mM EDTA DPBS.
  f. Incubate at 37°C for 4 min.
    **CRITICAL:** Do not leave the cells for longer than 5 min to avoid single cell suspensions. It is best to aim for small clumps of 4–20 cells, since iPSC require cell-cell contact to prevent spontaneous differentiation and allow growth in the absence of ROCKi.
  g. Observe cells at 10× magnification on a standard lab bright field microscope, the iPSC should still be attached but should now be rounded up. If not rounded, incubate for slightly longer.
  h. Remove 500 μL EDTA DPBS.
  i. Using a P1000 add 1 mL E8 to each well and detach the iPSC by pipetting up to a maximum of 5 times.
  j. Transfer the iPSC suspension to a 15 mL falcon containing 11 mL E8 media (final volume 12 mL).
  k. Remove vitronectin from a 6 well-plate (wp).
  l. Add 2 mL of cell suspension to each well of a vitronectin coated 6wp for a 1:6 split.
  m. Agitate in a cross-hatch method and incubate cells at 37°C 5% CO_2_.
6. 24 h post thaw check on the cells – they should be evenly distributed forming defined colonies.
7. Perform daily 100% media changes with 2 mL E8 until the cells reach 70%–80% confluency.

**CRITICAL:** In order to achieve good EB differentiation the iPSC must be kept below 90% confluent, with no obvious signs of spontaneous differentiation. If in doubt, stop, and re-thaw iPSC. An example image of iPSC confluency is shown in Figure S1.

**CRITICAL:** Each 96 well plate requires 6 × 10^5^ iPSC and will provide enough EB for one x T175 myeloid factory. Scale production for the number of factories required for enough cells to do the screen at high coverage.

***Note:*** A factory is defined as a tissue culture flask containing multiple EBs. These flasks will produce precursors over multiple months which can be used for iMGL differentiations.

8. Generate embryoid bodies from cultured iPSC.
  a. Prepare 2× EB media stock as outlined in (Table 1).
    ***Note:*** This will require ∼3.5 mL per 96 well plate of EB
  b. Prepare a 50 mL tube of 1× E8 media containing by diluting equal volume of 2× EB media with 1× E8 containing 10 μM Y-27632 ROCKi.
  c. Prepare 96wp low attachment U bottom plates by adding 200 μL DPBS to the outer most wells.
  d. Remove media from the iPSC with an aspirator.
  e. Wash with 1 mL PBS.
  f. Add 0.5 mL TrypLE express.
  g. Incubate at 37°C 5% CO_2_ for 5 min to lift cells.
  h. While incubating prepare a 50 mL tube containing 8 mL DPBS per well harvested.
  i. Lift cells by gentle pipetting and transfer to 50 mL tube with DPBS.
  j. Wash well with 1 mL DPBS and transfer to 50 mL tube.
  k. Centrifuge at 400 g for 5 min at room temperature.
  l. Resuspend pellet in 1 mL E8 containing ROCKi.
  m. Take 10 μL and combine with 90 μL DPBS and further dilute with 100 μL 0.4% Trypan blue (final dilution 20), and count cells using a hemocytometer or automatic cell counter.
    **CRITICAL:** Work quickly, and only proceed if the cell viability is >90%.
  n. Adjust cells to 2 × 10^5^ cells/mL in E8 containing 10 μM ROCKi.
  o. Add 1:1 (v/v) of 2× EB media to 2 × 10^5^ cells/mL cell stock to make a working stock of 1 × 10^5^ cells/mL in 1× EB media.
  p. Transfer cells to a sterile reservoir.
  q. Using a multichannel. Add 100 μL cell suspension to the central wells of the 96wp (total 60 wells, 1 × 10^4^ cells/well).
  r. Centrifuge plates at 100 g for 3 min at room temperature.
  s. Carefully transfer the plate to a bright field microscope and check cells have pelleted at the bottom of the well.
    **CRITICAL:** Be careful when transferring cells from the centrifuge to the incubator, the cells should remain at the bottom of the plate. If disturbed while transporting, resuspend the cells with a multichannel and re-centrifuge.
  t. Incubate at 37°C 5% CO_2_.
9. After 24 h check for EB formation. The EBs should be rounded, have defined edges, with minimal cell death around the outside. The media should also be red/peach not yellow.
10. 48 h after seeding prepare 1× EB media for a media change as outlined in (Table 2).
  a. Using a multichannel, remove 50 μL spent media and add 100 μL fresh EB media.
  b. Incubate at 37°C 5% CO_2_.
  c. Store unused media at 4°C.
11. 96 h after seeding perform another media change, using a multichannel, remove 50 μL spent media and add 100 μL fresh EB media.
12. 6 d post seeding EB are ready to be transferred to T175 (factories).
  a. Prepare Factory media as outlined in (Table 3).
    **CRITICAL:** Factory media remains stable for up to 4 weeks. Discard and prepare new media after the 4 week deadline has passed.
  b. Coat Corning T175 with 15 mL of 0.1% Gelatin for 1 hr.
  c. Aliquot 50 mL DPBS into 50 mL tube.
  d. Take plate of EBs from the incubator and check under the scope, take representative image at 10×.
  e. Using wide-bore 200 μL tips, resuspend EB by pipetting up and down twice using a multichannel.
  f. Transfer the EB to a sterile reservoir.
  g. Repeat until all 60 EB are transferred to the reservoir.
  h. Check the plate for any EB which were not transferred, using a single channel p200 transfer any remaining EB to the reservoir.
  i. Place a 40 μm cell-strainer over a 50 mL tube.
  j. Using a 5 mL stripette, transfer the EB from the reservoir to the cell strainer.
  k. Wash the reservoir with 5 mL DPBS and transfer remaining EB to the cell strainer.
  l. Wash EB using 5 mL DPBS.
  m. Prepare a 50 mL tube containing 20 mL Factory media.
  n. Invert the 40 μm cell-strainer over the 50 mL tube containing Factory media.
  o. Flush off EB into the factory media using a stripette containing 5 mL Factory Media.
  p. Repeat step o.
  q. Remove and discard cell-strainer.
  r. Take T175 containing 0.1% Gelatin H_2_O from the incubator.
  s. Remove 0.1% Gelatin H_2_O using aspirator.
  t. Using 25 mL stripette, resuspend the EB in the 30 mL factory media and transfer to the Gelatin Coated T175.
    **CRITICAL:** Ensure even distribution of EB across the plate, to do this perform several aggressive left/right, forward/backwards movements of the flask. Do not touch or move the factories until the first feed at day 5.
  u. Label flask with date, volume of media, and day 0.
  v. Incubate at 37°C 5% CO_2_.
  w. Repeat the above steps for the next set of plates.
13. Factory age d5.
  a. Warm Factory media to room temperature.
  b. Check on factories, EB should have adhered and have stromal skirts protruding out from the EB.
  c. Add 20 mL of factory media to each flask by inverting the flask and adding media slowly to the lid of the flask, this is to avoid disturbing the EB.
  d. Record date, media change, and total volume on the lid using an ethanol resistant pen.
  e. Incubate at 37°C 5% CO_2_.
14. Factory age d10.
  a. Warm Factory media to room temperature.
  b. Check on factories, EB should have adhered and have stromal skirts protruding out from the EB, iMGL precursors might begin to appear in the supernatant.
  c. Remove 25 mL of factory media from each flask by lifting and tipping the flask onto its corner, discard the media.
  d. Add 25 mL of factory media to each flask by inverting the flask and adding media slowly to the inside upper surface of the flask, this is to avoid disturbing the EB.
  e. Record date, media change, and total volume on the lid using an ethanol resistant pen.
  f. Incubate at 37°C 5% CO_2_.
15. Factory age d15.
  a. Warm Factory media to room temperature.
  b. Check on factories, EB should have adhered and have stromal skirts protruding out from the EB, iMGL precursors should now be appearing in the supernatant. An example image is in Figure S1.
  c. Place a 40 μm cell-strainer over a 50 mL tube.
  d. Remove 25 mL of factory media from each flask and strain cells, be careful not to remove EB from the media.
    **CRITICAL:** Volumes removed can vary dependent on the state of the factory and concentration of the iMGL precursors. We recommend removing a minimum of 50% of the total volume up to a maximum of 80%. Always replace the same volume of media that is removed.
  e. Add 25 mL of factory media to each flask by inverting the flask and adding media slowly to the lid of the flask, this is to avoid disturbing the EB.
  f. Record date, media change, and total volume on the lid using an ethanol resistant pen.
  g. Incubate at 37°C 5% CO_2_.
  h. Repeat media changes for all factories, combining harvests from 2 factories into one 50 mL tube. The iMGL precursors can be kept at room temperature during this procedure without deteriorating.
  i. Once all factories have been harvested, the filtered cells from d. can be centrifuged at 400 g for 5 min at room temperature.
  j. Remove the supernatant.
  k. Resuspend one pellet in 1 mL factory media using p1000, transfer resuspended cells to another pellet and resuspend again. Do this to combine all 50 mL tubes into one.
  l. Adjust to 40 mL using factory media.
  m. Dilute an aliquot of iMGL precursor cells with 0.4% trypan blue and calculate the concentration and viability using a hemocytometer or automatic cell counter.
  n. Adjust to 5 × 10^5^ cells/mL with factory media, cells can now be seeded in ITMG for pilot iMGL experiments if required.
16. Repeat the above steps every 4–5 d up until d40 when feeding can be reduced to every 7 d.

***Optional:*** QC the precursor quality by flow cytometry for markers of myeloid differentiation such as CD14, CD68, CD11b and a proliferation marker such as Ki67.

**CRITICAL:** iMGL precursors are most uniform from around d20-d40, we recommend not using cells which are harvested before or after these time points for critical experiments. They can however be used to generate pilot data.

**Table 1:** 2× embryoid body media

Reagent	Final concentration	Amount
E8	1×	50 mL
Y-27632	10 μM	50 μL
BMP-4	100 ng/mL	200 μL
SCF	40 ng/mL	200 μL
VEGF	100 ng/mL	200 μL

Store at 4°C for 1 week.

**Table 2:** 1× EB media for feeding

Reagent	Final concentration	Amount
E8	1×	50 mL
BMP-4	50 ng/mL	100 μL
SCF	20 ng/mL	100 μL
VEGF	50 ng/mL	100 μL

Store at 4°C for 1 week.

**Table 3:** 1× factory media

Reagent	Final concentration	Amount
X-VIVO 15	1×	500 mL
Glutamax	2 mM	5 mL
Β-mercaptoethanol	0.055 mM	500 μL
M-CSF	100 ng/mL	500 μL
IL-3	25 ng/mL	250 μL

Store at 4°C for 1 month.

### Large-scale production of iMGL precursors

**Timing: 20–30 days**

This section outlines two methods which can be utilized to increase the numbers of the iMGL precursors to the numbers required for CRISPR screening. We mention two different methods, either incremental increases of culture volume in the factory flasks, or the use of a low-speed bioreactor, to pool subsequence iMGL precursor harvests from multiple factories into one homogenous pool of cells.

**CRITICAL:** Scaling of iMGL precursor production can be done in two ways. Either repeatedly topping factories with additional media or pooling harvests into a slow speed bioreactor. We recommend the latter as this will remove batch variation across different harvests and will also pool different time points into a homogenous mixture of different ages. Through RNAseq analysis, the age of the precursors is a significant driver of variation within the dataset.

17. Topping factories with more media.
  ***Note:*** From factory age d21 you can begin the expansion as they should have undertaken a proliferative burst, this is a rapid expansion of the number of precursors within the media which happens at this early time point.
  a. Quality control the factories.
    i. Take 1 mL of supernatant from the factory and calculate the viability by combining 1:1 (v/v) with 1 mL 0.4% Trypan blue.
      ***Note:*** There should be high survival (>90% viability), if less than 90% do not proceed and feed as previous by removing 30 mL and replacing with 30 mL fresh factory media.
    ii. Calculate the concentration of iMGL precursors within each factory.
      ***Note:*** The optimal concentration should be between 2 × 10^5^ cells/mL and 5 × 10^5^ cells/mL.
      **CRITICAL:** Is the viability >90% and cell concentration within the factories < 5 × 10^5^ cells/mL? Continue to step 17b. If not, proceed with regular 50%–80% total media change with fresh factory media as outlined in Step 15.
  b. Proceed by adding 30–60 mL fresh factory media to make the final concentration of iMGL precursors between 1.5 × 10^5^ cells/mL and 2 × 10^5^ cells/mL.
    **CRITICAL:** The Corning flasks recommended by this protocol are deep enough to hold up to 150 mL total factory media. Take care when transporting to and from the incubator and tissue culture hood.
  c. Incubate at 37°C 5% CO_2_.
  d. Repeat the above for every feed until enough cells are available for the experimental requirements.
  e. Once enough cells are present, harvest up to 80% of the total volume of each flask and filter through a 40 μm cell strainer into a 50 mL conical tube.
  f. Replace media from with fresh factory media to a total volume of 60 mL.
  g. Centrifuge harvested cells at 400 g for 5 min at room temperature.
  h. Remove the supernatant with an aspirator.
  i. Resuspend the cell pellet in 5 mL factory media and combine all pellets into one tube or sterile bottle.
  j. Perform a cell count and viability check using 0.4% Trypan blue and a dilution of the cell stock.
  k. Continue with the screen or experiment.
18. Using a slow speed stirrer and bioreactor.
  a. From factory age d21 you can begin the expansion as they should have undertaken the proliferative burst.
  b. Harvest between 50%–80% of total media from the factories using a stripette.
  c. Filter the precursors through a 40 μm cell strainer into a 50 mL tube.
  d. Replace media from factories with fresh factory media to a total volume of 60 mL.
  e. Centrifuge harvested cells at 400 g for 5 min at room temperature.
  f. Remove the supernatant with an aspirator.
  g. Resuspend the cell pellet in 5 mL factory media and combine all pellets into one tube or sterile bottle.
  h. Quality control the precursors.
    i. Take 1 mL of supernatant from the factory and calculate the viability by combining 1:10 (v/v) with DPBS then 1:1 (v/v) with 0.4% Trypan blue (final dilution 1:20). There should be high survival (>90% viability), if less than 90% do not proceed.
    ii. Calculate the concentration of iMGL precursors and adjust to 5 × 10^5^ cells/mL with factory media.
      ***Optional:*** QC the precursor quality by flow cytometry for markers of myeloid differentiation such as CD14, CD68, CD11b and a proliferation marker such as Ki67.
      **CRITICAL:** The 1L bioreactor from Corning has a minimum working volume of 200 mL. This is approximately 14× T175 KOLF2.1J factories where 50% of the total supernatant containing iMGL precursors has been harvested. If you have less than 200 mL dilute the cells to make up the difference to a minimum concentration of 2 × 10^5^ cells/mL.
  i. Add precursors to the bioreactor and incubate at 37°C 5% CO_2_ at a constant speed of 30 rpm.
  j. Immediately prior to the next harvest perform a QC of the bioreactor.
    i. Take 1 mL of supernatant from the bioreactor and calculate the viability by combining 1:1 (v/v) with 0.4% Trypan blue. There should be high survival (>90% viability).
    ii. Calculate the concentration of iMGL precursors, this should be similar to the starting concentration of 5 × 10^5^ cells/mL.
      ***Note:*** During the first few harvests this may increase due to proliferation.
      ***Optional:*** QC the precursor quality by flow cytometry for markers of myeloid differentiation such as CD14, CD68, CD11b and a proliferation marker such as Ki67.
  k. Repeat steps 31–39 every 5 d.
  l. Before adding to the bioreactor ensure that the final concentration of cells in the bioreactor is adjusted to 5 × 10^5^ cells/mL.
  m. Ensure that the total volume of fresh media added to the bioreactor is at least 50% of the total final volume of the bioreactor. I.e. if the starting volume is 200 mL make sure you are adding at least 200 mL of fresh factory media.
  n. If the volume of fresh media added is less than 50% of the total volume prior to addition (in the above example this could be 150 mL), harvest cells from the bioreactor to make up the difference (50 mL). Centrifuge at 400 g for 5 min at room temperature. Remove supernatant and resuspend in an equal volume of fresh factory media. Add back to the bioreactor.
  o. Once enough cells are present for your screen, harvest up to 90% of the total volume of each bioreactor and filter through a 40 μm cell strainer into a number of 50 mL conical tubes.
  p. Centrifuge harvested cells at 400 g for 5 min at room temperature.
  q. Remove the supernatant with an aspirator.
  r. Resuspend each cell pellet in 5 mL factory media and combine all pellets into one tube or sterile bottle.
  s. Perform a cell count and viability check using 0.4% Trypan blue and a dilution of the cell stock.
  t. Continue with the screen or experiment.

### Differentiation of iMGL precursors to iMGL in ITMG

**Timing: 14 days**

This section outlines the final step of the differentiation from the iMGL precursor cells to defined iMGL. The following protocol provides the required media composition, cell numbers required for different formats, the feeding schedule, and example images.

**CRITICAL:** It is critical to use precursors which have gone through the proliferative burst, as a result we recommend that cells no younger than d21 are used for CRISPR screening.

19. Prepare ITMG microglial media as outlined in (Table 4).
20. Cells are seeded at 8.8 × 10^4^ cells/cm^2^, see (Table 5) for numbers for various tissue culture plastic, Greiner plastic is recommended as the media was developed using Greiner.
21. iMGL precursors should be in factory media and have been quantified.
22. Take required cell number and centrifuge at 400 g x 5 min at room temperature.
23. Remove supernatant with aspirator.
24. Resuspend in 1 mL ITMG with a p1000 to get a single cell suspension.
25. Adjust to required concentration with ITMG.
26. Add cells to vessel of choice.
27. Incubate at 37°C 5% CO_2_.
28. Replace 50% of the media at d3/d4 post seeding with fresh ITMG, discard spent media.

**CRITICAL:** The cells might be loosely adherent at this point, be careful when removing cells. Do this slowly to reduce the risk of losing cells. If cells are removed, keep the supernatant and centrifuge at 400 g for 5 min at room temperature, resuspend the pellet in fresh ITMG and add back into the culture vessel.

29. Replace 50% of the media at d7 post seeding with fresh ITMG, discard spent media.
30. Replace 50% of the media at d10/d11 post seeding with fresh ITMG, discard spent media.

**CRITICAL:** The cell morphology changes between d10 and d14 post seeding. The cells will become more polarized and “microglia like”.

31. Cells are ready for assays at d14 post seeding.

**Table 4:** 1× ITMG microglial media

Reagent	Final concentration	Amount
ADMEM/F12	1×	50 mL
Glutamax	2 mM	500 μL
IL-34	100 ng/mL	50 μL
TGFB1	50 ng/mL	50 μL
M-CSF	25 ng/mL	12.5 μL
GM-CSF	10 ng/mL	50 μL

Store at 4°C for 2 weeks.

**Table 5:** Recommended culturing densities and volumes for iMGL

Tissue culture vessel	Size (cm^2^)	iMGL cell number	Culture volume
96 well plate	0.34	3 × 10^4^	100–200 μL
48 well plate	1	8.8 × 10^4^	200–400 μL
24 well plate	1.9	1.67 × 10^5^	500 μL–1 mL
12 well plate	3.9	3.43 × 10^5^	1 mL–2 mL
6 well plate	9.6	8.45 × 10^5^	2 mL–4 mL
T25	25	2.2 × 10^6^	5–7 mL
T75	75	6.6 × 10^6^	15 mL
T175	175	1.54 × 10^7^	30 mL

### Large-scale production of lentivirus and VPX virus-like particles in 15 cm tissue culture plates

**Timing: 5 days**

This section outlines the successful production of VPX-VLPs and lentiviral CRISPR library used for screening in HEK293T cells. The protocol outlines the conditions required for producing high numbers of high quality virions. The protocol is identical for the production of either VPX-VLPs and the lentiviral CRISPR library, the only difference is the combinations of plasmids in the initial transfection which are outlined below.

For packaging the lentiviral library the following transfection into HEK293T is required: Lentiviral library vector pool, pMD2.G, psPAX2.

For preparing VPX-VLPs the following transfection into HEK293T is required: pSIV-D3psi/delta env/delta Vif/delta Vpr pMD2.G, psPAX2. These VPX-VLPs deliver the VPX protein which is required to enhance lentiviral transduction of the main CRISPR library through SAMHD1 mediated knockdown.

**CRITICAL:** Biosafety precautions: Proper handling of lentivirus should be followed as outlined by your institution’s Environmental Health and Safety Office including a specific risk assessments.

**CRITICAL:** Before starting it is vital to have high concentrations of the plasmid DNA required for transfection (>500ng/μl). Prepare plasmid DNA through maxiprep, we use the ZymoPURE II Plasmid Maxiprep Kit for all our preps, following manufacturer’s instructions.

***Note:*** The plasmids required for packaging both the VPX-VLPs or the lentiviral library are second generation lentiviral vectors. pMD2.G expresses the VSV-G envelope protein, whereas psPAX2 encodes the packaging proteins gag and pol. The lentiviral library is a pool of vectors each vector contains an individual guide RNA (sgRNA) or two guide RNA (dgRNA), Cas9, and puromycin selection.

32. Seed HEK293T.
  a. Coat 15 × 15 cm dishes with 15 mL 0.1% Gelatin solution for 1 h at 37°C.
  b. Remove gelatin coating.
  c. Plate 14 × 10^6^ HEK293T cells per 15 cm dish in 30 mL HEK293T media (Table 6).
  d. Incubate at 37°C, 5% CO_2_.
33. 18–24 h post seeding, transfect pSIV-D3psi/delta env/delta Vif/delta Vpr OR lentiviral Library, pMD2.G and psPAX2 into HEK293T.
  **CRITICAL:** Check the HEK293T cells, they should be approximately 70% confluent and an even monolayer. If not stop and discard as lentiviral production will be sub-optimal.
  a. Warm HEK293T media, OptiMEM, and plasmids to room temperature before starting.
  b. Prepare a mix of DNA as outlined in (Table 7), adding OptiMEM, then plasmids, then PLUS Reagent.
  c. Mix by inversions and wait 5 min at room temperature.
  d. Add 90 μL Lipofectamine LTX for each 15 cm plate required (1.35 mL for 15 plates) directly to the OptiMEM:DNA mixes.
  e. Mix by inversions and wait 30 min at room temperature.
  f. Remove media from plates.
  g. Add 7.5 mL of OptiMEM:DNA:Lipofectamine to each plate in dropwise motion.
  h. Add 7.5 mL OptiMEM to each plate, add at the side slowly.
  i. Incubate at 37°C, 5% CO_2_.
34. Replace media 18–24 h post transfection.
  a. Warm HEK293T media to room temperature before using.
  b. Carefully remove the transfection mixes with 25 mL stripette and discard.
  c. Slowly add 25 mL HEK293T media to each plate, try not to dislodge cells.
  d. Incubate at 37°C, 5% CO_2_.
35. First collection and concentration (48 h post transfection).
  a. Warm HEK293T media to room temperature before use.
  b. Cool a benchtop centrifuge with fixed rotor for 50 mL tubes to 4°C.
  c. Collect 20 mL of viral supernatant into 50 mL conical tubes, be careful not to aspirate any HEK293T as this will reduce subsequent viral titer.
  d. Replace with 20 mL of fresh HEK293T media and incubate at 37°C, 5% CO_2_.
  e. Harvest from all plates before centrifuging conical tubes at 500 g for 5 min to pellet any transferred HEK293T cells. Ensure you use sealed buckets to prevent aerosol generation.
  f. While centrifuging, attach a 0.45 μm 500 mL filter (VWR, 734–5068) to a sterile or autoclaved 500 mL bottle.
  g. Transfer the supernatant from e. into the reservoir attached to the 500 mL filter, and filter the supernatant under pressure.
  h. Transfer 45 mL of filtered viral supernatant into enough 50 mL conical tubes to fill the fixed bucket centrifuge and label. Any remaining viral supernatant can be stored at 4°C and used up to 24 h later.
    **CRITICAL:** Check the performance rating of the 50 mL conical tubes, some are not tested to speeds of 20,000 g.
  i. Spin at 20,000 g for 3 h at 4°C to pellet virions.
  j. The virions should appear as an opaque/off white pellet at the bottom of the conical tube. Mark the outside of the tube with an ethanol resistant pen when removing from the centrifuge.
  k. In the hood, remove the supernatant. If there is remaining filtered virus from step g/h. Add more viral supernatant to the tubes and repeat step i. If not proceed to step l.
    **CRITICAL:** Ensure to place the 50 mL conical tube back in the centrifuge in the same orientation.
  l. Remove as much media as possible from the conical tubes using a P1000, take care not to disturb the pellet.
  m. Add 250 μL 1.5% BSA/PBS to each tube and incubate pellet side down on ice for 1 h.
  n. After 1 h gently resuspend the pellets with a P1000 and combine into a single aliquot, store in a cryovial at 4°C overnight.
36. Second collection and Concentration (72 h post transfection).
  a. Repeat the above steps with the following modifications.
    i. Discard the plates once step 35.c is complete, do not feed again.
    ii. Resuspend the viral pellets in 125 μL 1.5% BSA/PBS in step 35.m.
  b. Once the second harvest has been resuspended combine with the first harvest stored at 4°C.
  c. Create small single use aliquots (approx. 200 μL) and store at −80°C.
37. Repeat from step 32 for the lentiviral CRISPR library.

**Table 6:** HEK293T media

Reagent	Final concentration	Amount
DMEM	1×	445 mL
Glutamax	2 mM	5 mL
FBS	10%	50 mL

**Table 7:** Transfection mixes for large scale lentiviral generation

Reagent	Volume (×1 15 cm)	Volume (×15 15 cm)
OptiMEM	7.5 mL	112.5 (make volume to 15 mL)
PLUS Reagent	30 μL	450 μL
pSIV-D3psi/delta env/delta Vif/delta Vpr OR Lentiviral Library plasmid pool	7.5 μg	112.5 μg
psPAX2 (packaging plasmid)	18.5 μg	277.5 μg
pMD2.G (VSVG envelope plasmid)	4 μg	60 μg

### VPX virus-like particle titration by western blotting

**Timing: 7 days**

This section outlines the protocol for titration of the VPX-VLPs in iMGL to determine the optimal concentration for the reduction of SAMHD1. This section utilizes western blotting to calculate the functional titer of the VPX-VLPs.

**CRITICAL:** Biosafety precautions: Proper handling of lentivirus should be followed as outlined by your institution’s Environmental Health and Safety Office.

**CRITICAL:** The age of the iMGL precursors and density will influence the transduction efficiency, with younger precursors (d14-28) being more sensitive to lentiviral toxicity than d28+. Fewer virions are required for a higher infectivity at d14 than d28.

38. Harvest iMGL precursors from either factory or bioreactor.
  a. Filter iMGL through a 40 μm EasyStrainer into a 50 mL conical tube.
  b. Spin at 400 g for 5 min to pellet the cells.
  c. Resuspend the cells in room temperature ITMG.
  d. Count the cells.
  e. Adjust concentration to 3.43 × 10^5^ cells/mL in ITMG.
  f. Add polybrene to a final concentration of 4 μg/mL.
  g. Add 1 mL of cells (3.43 × 10^5^ cells) to each well of a 2 × 12 well plates.
39. Thaw aliquot of VPX-VLPs at 37°C.
  a. Add VPX-VLPs to duplicate wells in the following dilution series.
    i. 0.625 μL 1:1600
    ii. 1.25 μL 1:800
    iii. 2.5 μL 1:400
    iv. 5 μL 1:200
    v. 10 μL 1:100
    vi. 20 μL 1:50
    vii. 40 μL 1:25
  b. Include 2 wells of non-transduced negative control wells.
  c. Mix by gentle agitation.
  d. Incubate at 37°C, 5% CO_2_.
40. Remove media 18–24 h post transduction.
  ***Note:*** The cells will be loosely attached, take care when remove the media to do it slowly to prevent loss of cells.
  a. Remove the media with a P1000.
  b. Add 1 mL fresh ITMG media.
  c. Incubate at 37°C, 5% CO_2_.
41. Harvest protein for western blotting.
  a. 72 h post transduction gently remove the spent media and wash each well twice with 1 mL cold PBS to remove dead cells.
  b. Add 100 μL ice cold RIPA containing protease inhibitors to each well.
  c. Incubate on ice for 5 min.
  d. Transfer the lysate to an ice cold 1.5 mL tube.
  e. Centrifuge at 5000 g for 5 min at 4°C to pellet any debris.
  f. Transfer the supernatant to a fresh 1.5 mL tube and move directly to protein quantification or store at −80°C.
42. Quantify the protein content in the samples using a BCA assay, following the manufacturer’s instructions.
43. Western blotting for SAMHD1 and GAPDH.
  a. Run 10–40 μg of total protein per samples by SDS-PAGE electrophoresis on a 4%–8% TRIS Acetate or 4%–12% Bis-TRIS plus acrylamide gel for 75 min at 100 V in running buffer.
  b. Transfer the proteins to a PVDF membrane activated with 100% methanol for 5 min using wet transfer at 75 min at 100 V in transfer buffer on ice.
  c. Block the membrane with 5% skimmed milk powder (w/v) in PBST for 1 h on a lab rocker at room temperature.
  d. Remove the block and wash the membrane 6 × 5 min with 1× PBST on the lab rocker at room temperature.
  e. Incubate the membrane with primary antibodies made up in 1% skimmed milk PBST overnight on a lab rocker at 4°C.
    i. SAMDH1 Antibody – Dilute 1:500.
    ii. GAPDH Antibody – Dilute 1:1000.
  f. Remove the primary antibody and wash the membrane 6 × 5 min with 1× PBST on the lab rocker at room temperature.
  g. Incubate the membrane with secondary antibodies made up in 5% skimmed milk PBST on a lab rocker at room temperature.
    i. Rabbit Anti-mouse IgG (Light Chain Specific) (D3V2A) mAb (HRP Conjugate) – Dilute 1:2500.
    ii. Anti-rabbit IgG, HRP linked antibody – Dilute 1:2500.
  h. Remove the secondary antibody and wash the membrane 6 × 5 min with 1× PBST on the lab rocker at room temperature.
  i. Incubate with 2 mL HRP substrate for 3 min at room temperature protected from light.
  j. Visualize chemiluminescence signal using transilluminator of choice.
  k. Normalize SAMHD1 expression to GAPDH expression.
  l. Normalize the SAMHD1 as relative to the control.

**Figure 2:**
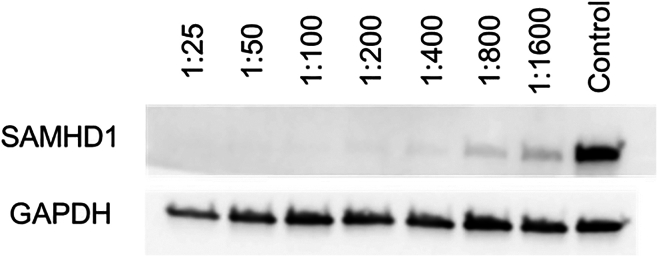
Example western blots for titration of VPX-VLPs by examining SAMHD1 knockdown GAPDH is used as a loading control. A 1:400 dilution of VPX-VLPs results in sufficient knockdown for lentiviral transduction of iMGL.

***Note:*** Using a dilution between 1:800 and 1:400 usually results in full knockdown of SAMHD1 (Figure 2).

### Titration of CRISPR libraries by puromycin selection

**Timing: 7 days**

This section outlines the protocol for titration of the lentiviral CRISPR libraries in iMGL to determine the optimal MOI for the screen. This section utilizes puromycin selection followed by a resazurin assay to calculate the functional titer of the lentiviral CRISPR libraries based on cellular survival post transduction and selection.

**CRITICAL:** Biosafety precautions: Proper handling of lentivirus should be followed as outlined by your institution’s Environmental Health and Safety Office.

**CRITICAL:** The multiplicity of infection (MOI) must be determined under the same conditions as used for the screen, this includes the manufacturer of the tissue culture plastic, media constituents, cell line, plating density, and the viral preparation.

**CRITICAL:** The age of the iMGL precursors and density will influence the transduction efficiency, with younger precursors (d14-28) being more sensitive to lentiviral transduction than d28+. Meaning that fewer virions are required for a higher infectivity than d28 so you will observe increased lentiviral toxicity. We recommend testing your lentivirus prep on d28 cells.

***Note:*** This method utilizes 96wp format for titration, these cannot be reliably scaled to larger formats, so always include a selection control in your screen to confirm the true MOI.

**Figure 3:**
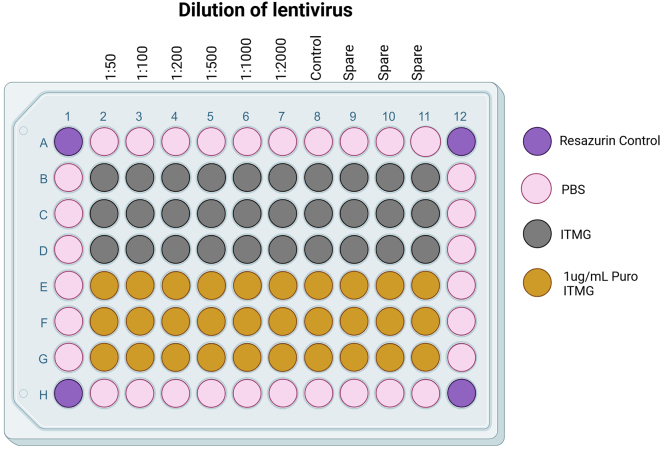
Example plate layout for titering lentiviral libraries Serial dilutions of virus from columns 2:6, including non-transduced controls in column 7. Outside wells are filled with media to prevent evaporation. Gray Rows B:C contain ITMG without puromycin, Yellow Rows E:F have 2 μg/mL puro ITMG added at D3 (final concentration 1 μg/mL). The purple corner wells contain 200 μL PBS plus 20 μL resazurin to act as a normalizing control.

44. Harvest iMGL precursors from either factory or bioreactor.
  a. Centrifuge at 400 g for 5 min to pellet the cells.
  b. Resuspend the cells in room temperature ITMG.
  c. Count the cells.
  d. Adjust concentration to 3 × 10^5^ cells/mL in ITMG.
45. Add polybrene to a final concentration of 4 μg/mL.
46. Thaw a vial of VPX-VLPs at 37°C.
  a. Add VPX-VLPs at the concentration determined from the SAMHD1 western blot.
  b. Mix by inversions.
47. Aliquot 100 μL cells/polybrene/VPX-VLPs into wells of a 96 well plate, plate layout is provided in Figure 3.

***Note:*** Exclude the outer wells and fill with PBS

**Figure 4:**
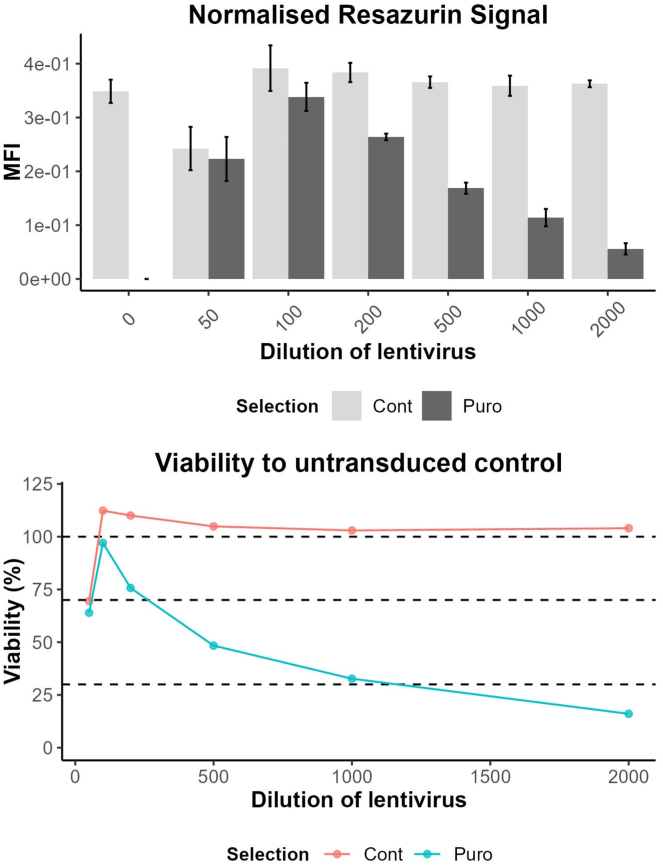
Example titration plots from a lentiviral library titered with resazurin (Alamar blue) The average mean fluorescent intensity (MFI) for each dilution selected with 1 μg/mL puromycin (Puro) or the matched control (Cont), and the normalized viability % as compared to the non-selected control. Both methods are informative for measuring the correct titer. A 1:50 dilution, while having high transduction efficiency of nearly 100% reduces the cell count through cytotoxic effects as the non-selected cells have only 75% viability compared to the control. For this example, a 1:1000 dilution of lentivirus would give an MOI of 0.3 required for single integrations. Error bars are mean +/− standard deviation.

48. Thaw a vial of lentiviral library to be tired at 37°C.
  a. Add lentivirus to 6 wells in the following dilution series.
    i. 2 μL 1:50
    ii. 1 μL 1:100
    iii. 0.5 μL 1:200
  b. Dilute lentivirus stock 1:10 in ITMG media then prepare the next set of dilutions.
    i. 2 μL 1:500
    ii. 1 μL 1:1000
    iii. 0.5 μL 1:2000
  c. Include 6 wells of non-transduced negative control wells.
  d. Mix by gentle agitation.
  e. Incubate at 37°C, 5% CO_2._
49. Remove media 18–24 h post transduction.
  ***Note:*** The cells will be loosely attached, take care when remove the media to do it slowly to prevent loss of cells.
  a. Remove the media with multichannel.
    ***Note:*** Move from the lowest concentration to the highest.
  b. Add 100 μL fresh ITMG media.
  c. Incubate at 37°C, 5% CO_2_.
50. 72–96 h post transduction select the cells with puromycin.
  ***Note:*** Half of the wells are selected with puromycin and half are with ITMG only, this is to check the toxicity of the virus at high doses.
  a. Prepare a 2× stock of puromycin in ITMG (2 μg/mL).
    ***Note:*** Puromycin concentrations can be optimized using a kill curve. We have found a final concentration of 1 μg/mL for 72 h enough to kill non-transduced KOLF2.1J.
  b. Add 100 μL of a 2× stock of puro/ITMG (2 μg/mL)to 3 of the wells per dilution for a final working concentration of 1 μg/mL.
  c. Add 100 μL ITMG only to the remaining 3 wells of each dilution.
  d. Incubate at 37°C, 5% CO_2_.
51. Check cells over the next few days to confirm that the selection is working, the non-transduced cells should be 100% dead with puro selection, and the non-selected wells should be 100% viable.
52. 7 days post transduction the cells are ready for viability testing with resazurin.
  a. Prepare a 1:100 dilution of resazurin from 10 mg/mL stock.
  b. Add 20 μL to each well (final dilution 1:1000).
  c. Include corner wells containing 200 μL PBS and 20 μL resazurin with no cells to normalize the data.
  d. Incubate at 37°C for 1–4 h if measuring fluorescence, or 24 h if measuring absorbance.
  e. Read fluorescence or absorbance at room temperature.
    i. Fluorescence: Excitation 540–570 m, peak excitation 570 nm, Emission peak 585 nm.
    ii. Absorbance: 570 nm using 600 nm as reference wavelength.
  f. Normalize the results to the empty wells to remove the background fluorescence.
  g. Normalize the results to the non-transduced cells non-selected to give a % survival (Figure 4).
  h. Determine the virus volume which gives 30%–40% survival with selection vs without.
  i. This is the volume required for an MOI of 0.3.
  j. In the example in Figure 4 a 1:1000 dilution of the virus would provide an MOI ∼0.3.

***Note:*** At higher concentrations of lentivirus the survival will drop below the non-selected control, this is because the lentivirus is toxic at these high concentrations. This is seen at the 1:50 dilution in Figure 4, as the number of non-selected control cells is lower than the non-transduced control.

### Large-scale CRISPR screening in iMGL

**Timing: 15 days**

This section outlines how to undertake a pooled FACS based CRISPR screen for phagocytosis based on the prior generation of iMGL precursor cells, successful titration of the VPX-VLPs and lentiviral CRISPR libraries. The protocol defines the transduction, required control conditions, how to lift the cells for FACS, light fixation with PFA, recommended gating strategy, and pelleting of cells for subsequent processing.

**CRITICAL:** Biosafety precautions: Proper handling of lentivirus should be followed as outlined by your institution’s Environmental Health and Safety Office.

***Note:*** Each T175 can support 15.4 × 10^6^ cells total. Adjust the number of flasks accordingly for the correct coverage. See Table S1 for a helpful calculator of cell numbers required. With fixation at the end of the 14 d expect to retrieve approx. 20% of the input number of cells. With sorting and DNA extraction this can reduce to approx. 2% of the total starting population. We recommend fixing the cells at the end of the assay as phenotypes can change during the long sorting times.

***Note:*** We recommend including the following controls, 2 × T175 to collect as a baseline T0 to check lentiviral library composition. 2 × T175 as selection controls, one transduced with the library and one non-transduced, to calculate the actual MOI. 1 × T175 as a transduction control for flow cytometry, 1 × T175 as a non-transduced control. And any assay specific controls required.

**CRITICAL:** Ensure the precursors have had enough time in the bioreactor to equilibrate, this will reduce the age effect of the different harvests. We recommend harvesting cells the day before screening into the bioreactor to allow at least 18 – 24 h for the cells to stabilize.

53. Harvesting precursors from bioreactor.
  a. Collect 5 mL of cells from the bioreactor.
  b. Dilute 1:1 with 0.4% Trypan blue.
  c. Count cells.
    ***Note:*** Do several counts for an accurate measurement, repeat if counts are out by 10%
  d. Harvest the number of cells required for screening into 50 mL conical tubes.
  e. Centrifuge at 400 × *g* for 5 min at room temperature to pellet.
  f. Remove media using aspirator.
  g. Resuspend cells in 10 mL factory media.
  h. Combine all resuspended cells into one homogenous mix.
  i. Count cells.
    ***Note:*** Do several counts for an accurate measurement, repeat if counts are out by 10%
  j. Centrifuge required cells at 400 g for 5 min at room temperature.
  k. Remove factory media using aspirator.
  l. Adjust cells to a final concentration of 7.7 × 10^5^ cells/mL in ITMG.
    **CRITICAL:** Cell density can influence phenotype, this has been optimized to provide enough cells following transduction and the first media change.
    ***Note:*** Each T175 requires 20 mL of ITMG media.
54. Add polybrene to a final concentration of 4 μg/mL, and mix by inversions.
55. Add dilution of VPX-VLPs which results in knockdown of SAMHD1 as determined by western blot titer, and mix by inversions.
56. Add dilution of CRISPR library which results in MOI of 0.3 if selecting cells with puromycin or MOI 1.5 if selection not required.

***Note:*** Selection could influence the outcome of the measured phenotype. Separate assays must be done prior to confirm or deny. This is left to the discretion of the user.

57. Mix by inversions.
58. Aliquot 20 mL of transduction mixture into a Greiner T175 flask.

***Note:*** Prepare non-transduced cells in the same manner, but do not include the CRISPR-library.

59. Allow cells to settle at room temperature for 5 min in the hood.
60. Incubate at 37°C 5% CO_2_ for 18–24 h.
61. 18–24 h post transduction observe the cells under the microscope.
  a. Carefully remove transduction media from each flask into a 50 mL conical tube.
  b. Replace transduction media with 20 mL fresh ITMG media.
    ***Note:*** To avoid disturbing the cells, invert the flask and add the media to the inside upper surface of the flask.
  c. Centrifuge the harvested transduction media at 400 g for 5 min to pellet the removed cells.
  d. Remove the transduction media with an aspirator.
  e. Resuspend the pelleted cells in 10 mL ITMG.
  f. Add the cells back to the corresponding flask.
    ***Note:*** Add the cells to the base of the flask very slowly as to not disturb loosely attached cells.
62. Incubate at 37°C 5% CO_2_.
63. 96 h post transduction the cells are ready for harvesting the T0 transduction reference, optional puro selection, and the first media change.
64. Harvest cells for T0 library collection to check initial representation of the library.
  ***Note:*** Ensure enough cells have been seeded for at least 200× coverage of the library. From the two T175 you can expect approx. 3 × 10^6^ cells worth of DNA following DNA extraction.
  a. Remove media using aspirator.
  b. Add 5–7 mL TrypLE Express.
  c. Incubate at 37°C 5% CO_2_ for 5 min before checking under the scope for detachment, if cells are not detached leave for longer, up to 10 min.
    ***Note:*** Cells will attach together and form a string-like mass, this is normal
  d. Collect cells into a 50 mL conical tube containing 20 mL PBS.
  e. Wash flasks with 10 mL PBS to collect any remaining cells.
  f. Centrifuge at 500 g for 5 m at room temperature to pellet cells.
  g. Remove PBS/TrypLE mix with aspirator.
  h. Store pellet at −80°C until DNA extraction.
65. Puro selection.
  ***Note:*** In order to ascertain an accurate MOI it is recommended to select two flasks (one transduced, another non-transduced) with 1 μg/mL puromycin.
  a. Prepare a stock of 2 μg/mL puromycin in ITMG.
  b. Remove 15 mL of media from flasks requiring selection.
  c. Replace with 15 mL 2 μg/mL puromycin ITMG (final concentration 1 μg/mL).
  d. Incubate at 37°C 5% CO_2_ until d 7 media change.
66. 50% media change of remaining flasks.
  a. Remove 15 mL of media from each flask.
  b. Replace with 15 mL of fresh ITMG.
    ***Note:*** Add media to the lid of the flask to prevent dislodging iMGL.
  c. Incubate at 37°C 5% CO_2_.
67. 7 d post transduction check the puro selected flasks for viability and perform a 50% media change of all flasks.
68. Puromycin checks; under the microscope check if cells are still viable in the non-transduced flask. If so replace 50% of the media with fresh 1 μg/mL puromycin ITMG.
69. 50% media changes of remaining flasks.
  a. Remove 15 mL of media from each flask.
  b. Replace with 15 mL of fresh ITMG.
    ***Note:*** Add media to the lid of the flask to prevent dislodging iMGL.
  c. Incubate at 37°C 5% CO_2_.
70. 10 d post transduction, perform the final 50% media change.
  a. Remove 15 mL of media from each flask.
  b. Replace with 15 mL of fresh ITMG.
    ***Note:*** Add media to the lid of the flask to prevent dislodging iMGL.
  c. Incubate at 37°C 5% CO_2_.
71. 14 d post transduction the cells are ready to perform your phenotypic assay of choice.
72. Harvesting and fixing cells post assay for FACS.
  a. Prepare a solution of 0.04% BSA/PBS.
  b. Check confluency of cells under the scope and record in notebook.
  c. Remove media with aspirator.
  d. Add 5 mL TrypLE.
  e. Incubate 37C 5% CO_2_ for 5 min before checking under the scope, tap to dislodge.
    ***Note:*** Cells will attach together and form a string-like mass, this is normal.
  f. Harvest cells from 3 or 4 × T175 into a 50 mL conical tube containing 20 mL 0.04% BSA/PBS.
  g. Wash out flasks using 10 mL of 0.04% BSA/PBS.
  h. Centrifuge 400 × *g* for 5 min to pellet.
  i. Resuspend in 1 mL 0.04% BSA/PBS.
    ***Note:*** Ensure at this stage there is a single cell suspensionAdjust to 20 mL with 19 mL 0.04% BSA/PBS
  j. Add 20 mL of 4% PFA and mix with stripette, final concentration is 2% PFA.
  k. Incubate at room temperature for 10 min to fix the cells.
  l. Mix every 2 m with 5 mL stripette to ensure a single cell solution remains.
  m. Centrifuge 400 g x 5 min to pellet.
  n. Remove PFA.
  o. Resuspend pellet in 5 mL 0.04% BSA/PBS with 5 mL stripette.
  p. Centrifuge 400 g x 5 m to pellet.
  q. Remove 0.04% BSA/PBS.
  r. Resuspend pellet in 5 mL 0.04% BSA/PBS with 5 mL stripette.
  s. Centrifuge 400 g x 5 min to pellet.
  t. Remove 0.04% BSA/PBS.
  u. Resuspend in 5 mL 0.04% BSA/PBS.
  v. Count cells using hemocytometer and adjust to 5 × 10^6^ cells/mL with 0.04% BSA/PBS.
73. Take approx. 1/5^th^ of the total volume as an unsorted population to use as a reference.
  a. Centrifuge at 400 g x 5 min room temperature.
  b. Remove BSA/PBS.
  c. Store pellet at −80°C until DNA extraction.
74. Store remaining cells at 4°C overnight.
75. The following day, filter cells through a FACS tube strainer and perform FACS as required, taking at least the top and bottom 20% of cells dependent on your assay.

**CRITICAL:** Sorting for 20% fractions of assay activity balances the enrichment of phenotypic extremes while maintaining sufficient cell numbers for downstream processing and representation. Further sorting of the two middle populations can also improve the coverage as described in.^15^

***Note:*** Precoat collection tubes and FACS tubes with 0.04% BSA/PBS.

76. Following FACS transfer sorted cells from collection tubes into 15 mL conical tubes.
  a. Wash collection tubes with 1 mL 0.04% BSA/PBS to collect any remaining cells.
  b. Centrifuge at 4000 g for 5 min to pellet the cells.
  c. Remove supernatant.
77. Store at −80°C until DNA extraction.

### DNA extraction

**Timing: 3 days**

This section outlines the successful extraction of DNA from the population of cells sorted following FACS, and from the control populations collected during the screen itself.

***Note:*** This section is split into high number (T0 or Unsorted populations >5 × 10^6^ total cells) or low number of cells (Sorted populations <5 × 10^6^ total cells). This assumes the pellets have been stored at −80°C

**CRITICAL:** The efficiency of the DNA extraction is critical to maintain library coverage. It is recommended to practice the DNA extraction on non-precious samples prior to the screening samples.

78. Thaw pellets on ice.
79. Centrifuge at 1200 g for 5 min at room temperature to pellet cells.
80. Remove as much supernatant as possible with a P1000 without dislodging the cell pellet.
81. Add Puregene cell lysis solution to each pellet.
  a. For high number cells add 6.6 mL per pellet.
  b. For low number cells add 300 μL per pellet.
82. Vortex to break up the cell pellet.
83. Add a 1:200 dilution of proteinase K.
  a. For high number cell pellet add 33 μL.
  b. For low number cell pellet add 1.5 μL.
84. Vortex to mix.
85. Incubate at 55°C overnight to improve de-crosslinking of fixed cells.
86. Take samples and cool to room temperature.

***Note:*** Solutions should be clear which is indicative of successful lysis

87. Briefly pulse centrifuge.
88. Add 1:200 RNAse A.
  a. For high number cell pellet add 33 μL.
  b. For low number cell pellet add 1.5 μL.
89. Incubate at 37°C for 15 min to 1 h.
90. Cool samples on ice for 1 min.
91. Add protein precipitation solution.
  a. For high number cell pellet add 2.2 mL.
  b. For low number cell pellet add 100 μL.
92. Vortex immediately for 20 s, samples should turn cloudy.
93. Centrifuge at 16,000 g for 3 min at 4°C to pellet protein.

***Note:*** If protein pellet is loose re-centrifuge

94. Prepare tubes with Isopropanol for DNA precipitation.
  a. For high number cell use 6.6 mL isopropanol in a 15 mL conical tube.
  b. For low cell number use 300 μL isopropanol containing 1 μL of glycoblue in 1.5 mL tube.

***Note:*** Do not use lo-bind tubes for this step

95. Transfer supernatant from step 93 into a tube containing isopropanol, taking care not to carry over the protein pellet.
  a. For high number cells transfer approximately 6.6 mL.
  b. For low number cells transfer approximately 300 μL.
96. Mix by inversions 50 times – DNA should begin to precipitate out of solution.
97. Incubate tubes at −20°C for 15 min.
98. Centrifuge at 20,000 g for 15 min at 4°C.
99. Remove supernatant into a tube labeled WA – keep at 4°C until DNA quantified.
100. Add 70% Ethanol to the DNA pellet.
  a. For high number cells add 1 mL.
  b. For low number cells add 300 μL.
101. Centrifuge at 16,000 g for 15 min at 4°C.
102. Remove supernatant into a tube labeled WB – keep at 4°C until DNA quantified.
103. Centrifuge at 7,000 g for 1 min at room temperature to pellet remaining 70% ethanol.
104. Remove remaining 70% ethanol using a P200 or P20.
105. Air dry the pellets at room temperature for 5 min with the lids opened or at 37°C in a heat block.

***Note:*** Do not over dry the pellet, pellets should go from white to colorless.

106. Resuspend the DNA in DNA hydration solution.
  a. For high number cells add 500 μL.
  b. For low number cells add 50 μL.

***Optional:*** DNA can be resuspended in nuclease-free water. This is recommended if an extra DNA precipitation is expected to avoid salt carry over.

107. Incubate at 65°C for 1 h shaking at 225 rpm.
108. Remove and store overnight at 4°C.
109. Quantify using Qubit High Sensitivity dsDNA kit.
  a. Once quantified, discard WA and WB tubes.

***Note:*** If low DNA quantity, DNA might remain in the WA or WB tubes. Adjust to 0.3 M Sodium Acetate and repeat precipitation.

110. Store DNA long term at −20°C.

### Library preparation

**Timing: 3 days**

This section describes the process of library preparation for Illumina sequencing of the gRNA sequences from the extracted DNA. First it describes the PCR conditions for amplifying the integrated gRNA cassettes, then a second round of PCR to add Illumina Indexes and barcodes. The total mass of DNA to be amplified to maintain coverage is dependent on library size, assuming 6 pg of DNA per diploid cell, you would require 1.2 ng of DNA input per gRNA in the library. For more information on minimal DNA input see.^16^ Table S1 contains the minimal mass of DNA required for PCR 1 as defined by the user library size, and required coverage.

**CRITICAL:** The library preparation outlined below was optimized for Illumina NovaSeq. Therefore the adapter sequences used in the second round PCR primers may not be applicable to your sequencing supplier. Please check before undertaking the indexing. We have provided PCR1 primers for either the all-in-one CRISPR sgRNA v3 backbone or the all-in-one CRISPR dgRNA v3 backbone. All library preparation should be undertaken in a dedicated room/hood to prevent cross-contamination.

***Note:*** We recommend checking that the primers can amplify the samples by performing a test batch of PCR using 1 μg transduced DNA per 50 μL reaction, taking samples every cycle from cycle 20 onwards. This is to confirm that the library is not over amplified in the first step.

111. Prepare primers for PCR1 – amplification of the sgRNA.
  a. Reconstitute all primers at 100 μM in Nuclease Free Water.
  b. Create a 10 μM working stock by diluting 10 μL of 100 μM stock in 90 μL Nuclease Free Water.
  c. Store at −20°C.
112. Set up enough PCR reactions to amplify enough DNA to maintain the minimal 200× coverage per gRNA as outlined in (Table 7).

***Note:*** Prepare master mix in a 15 mL or 50 mL conical tube and aliquot into a 96 well PCR plate before adding DNA.

***Note:*** Prepare a “water control” reaction not containing DNA

113. Seal plate and flick to mix.
114. Pulse centrifuge to remove bubbles.
115. Run on the PCR 1 program outlined in (Table 8) for a minimum of 20 cycles (or more if the optional PCR optimization step was carried out).

***Note:*** The number of cycles will be lower for the T0 and unsorted populations compared to the sorted populations.

**Figure 5:**
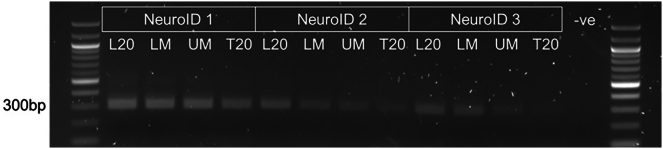
Example PCR1 for sorted populations from three independent repeats of a CRISPR screen (NeuroID 1, NeuroID 2, NeuroID 3) Ran for 25 cycles, note faint bands in all NeuroID 1 samples, some faint bands in NeuroID 2 and NeuroID 3, these were rerun for another 2 cycles. Note this is a dual-guide system. If using a single-guide system the band size would be ∼800 bp.

116. After PCR samples can be stored at 4°C overnight or −20°C for long term storage.
117. Run 2 μL of each sample on a 2% Agarose TAE gel for 30 min at 110 V to confirm amplification.
118. A single band should be present at approximately:
  a. 851 bp – for all-in-one CRISPR sgRNA v3 backbone.
  b. 305 bp – for all-in-one CRISPR dgRNA v3 backbone (Figure 5).

***Note:*** A faint band should be present, if not run the PCR for another 2 cycles and check again.

119. If band present, pool all PCR reactions from the same sample together in a 15 mL conical tube and vortex.
120. Aliquot into single use and store at −20°C long term or move directly on to the indexing PCR.

**CRITICAL:** The indexing PCR outlined below contains adapter sequences for Illumina sequencing using either MiSeq, HiSeq, or NovaSeq. If using a different sequencing supplier stop and confirm if the indexes will work with your pipeline.

121. To improve library complexity, pool the PCR2 10 μM F01 – F09 primers at an equimolar ratio in Nuclease Free Water, and vortex to mix.

***Note:*** Each sample will require a unique single index, this protocol has optimized 18 reverse primers, this should be enough for 3 repeats, with 4 sorted populations, and a T0 and Unsorted population for your screen. The indexes are outlined in (Table 9) and the full primer sequences are in the supplementary tables

***Note:*** We use single indexing, confirm with your sequencing supplier if this compatible.

**Figure 6:**
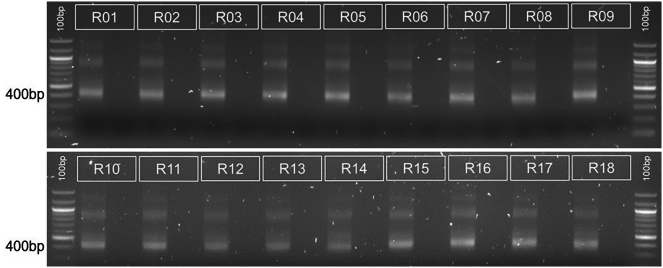
Indexing PCR2 for 10 cycles shows indexing band running at 400 bp in all samples This is the band to extract for library preparation.

122. In lo-bind 1.5 mL tubes, prepare master mixes with unique reverse primers as outlined in (Table 10), each sample requires 4 reactions.
123. Aliquot 45 μL of master mix into 4 wells of a 96 well plate per sample to index, including 1 well as a negative control.
124. Aliquot 5 μL of pooled PCR 1 product to each corresponding well.
125. Aliquot 5 μL of the water control PCR 1 product to the negative control well.
126. Repeat for each sample.
127. Seal and flick to mix.
128. Pulse centrifuge to remove bubbles.
129. Run on the PCR 2 program outlined in (Table 11) for 10 cycles.
130. After PCR samples can be stored at 4°C overnight or −20°C for long term storage.
131. Run 2 μL of each sample on a 2% Agarose TAE gel for 30 min at 110 V to confirm amplification.
132. A single band should be present at approximately 364 bp (Figure 6).

***Note:*** A faint band should be present, sometimes it can run at 400bp

133. If band present, pool all PCR reactions from the same sample together in a 1.5 mL tube vortex.
134. Store at −20°C long term or move directly on to gel extraction.

**Table 8:** Master mix for sgRNA or dgRNA barcode amplification

Reagent	Amount
NEBNext Ultra 5 Q5	25 μL
PCR 1 Forward Primer	2.5 μL
PCR 1 Reverse Primer	2.5 μL
DNA	Variable
Nuclease Free Water	Variable (to 50 μL)

**Table 9:** PCR 1 cycling conditions for sgRNA barcode amplification

Step	Temp	Time	Cycles
Initial Denaturation	98°C	30 sec	1
Denaturation	98°C	10 sec	20–30 cycles
Annealing	64°C	30 sec	
Extension	72°C	30 sec	
Final Extension	72°C	2 min	1
Hold	10°C	Forever

**Table 10:** Recommended indexing PCR 2 primer combinations

Sample	Repeat	Population	Forward primer	Reverse index primer
Screen 1 T0	1	Baseline	Pool PCR2 Forward	PCR2 Index 1
Screen 1 UN	1	Unsorted	Pool PCR2 Forward	PCR2 Index 2
Screen 1 L20	1	Sorted: Lowest 20%	Pool PCR2 Forward	PCR2 Index 3
Screen 1 LM	1	Sorted: Lower-middle 20%	Pool PCR2 Forward	PCR2 Index 4
Screen 1 UM	1	Sorted: Upper-middle 20%	Pool PCR2 Forward	PCR2 Index 5
Screen 1 T20	1	Sorted: Top 20%	Pool PCR2 Forward	PCR2 Index 6
Screen 2 T0	2	Baseline	Pool PCR2 Forward	PCR2 Index 7
Screen 2 UN	2	Unsorted	Pool PCR2 Forward	PCR2 Index 8
Screen 2 L20	2	Sorted: Lowest 20%	Pool PCR2 Forward	PCR2 Index 9
Screen 2 LM	2	Sorted: Lower-middle 20%	Pool PCR2 Forward	PCR2 Index 10
Screen 2 UM	2	Sorted: Upper-middle 20%	Pool PCR2 Forward	PCR2 Index 11
Screen 2 T20	2	Sorted: Top 20%	Pool PCR2 Forward	PCR2 Index 12
Screen 3 T0	3	Baseline	Pool PCR2 Forward	PCR2 Index 13
Screen 3 UN	3	Unsorted	Pool PCR2 Forward	PCR2 Index 14
Screen 3 L20	3	Sorted: Lowest 20%	Pool PCR2 Forward	PCR2 Index 15
Screen 3 LM	3	Sorted: Lower-middle 20%	Pool PCR2 Forward	PCR2 Index 16
Screen 3 UM	3	Sorted: Upper-middle 20%	Pool PCR2 Forward	PCR2 Index 17
Screen 3 T20	3	Sorted: Top 20%	Pool PCR2 Forward	PCR2 Index 18

**Table 11:** PCR 2 master mix

Reagent	Amount
NEBNext Ultra 5 Q5 MM	25 μL
Pool PCR2 Primers	2.5 μL
PCR2 Reverse	2.5 μL
PCR 1 Product	5 μL
Nuclease Free Water	15 μL

### Gel extraction

**Timing: 1 day**

This section outlines the protocol for the isolation of the required PCR product for sequencing through agarose gel electrophoresis, band excision, and gel extraction.

135. If samples stored at −20°C thaw on ice.
136. Prepare a 2% TAE Agarose gel with large wells to accommodate approx. 200 μL of sample. If using a standard gel comb, tape 4 wells together using autoclave tape.
137. To samples vortex and add 40 μL 6× loading dye purple (no SDS), flick to mix and briefly pulse centrifuge.
138. Load ∼240 μL into each well.

**CRITICAL:** Ensure to leave an empty well between samples to prevent cross contamination when extracting DNA.

139. Run gel at 60 V for 90 min, load 5 μL 100 bp ladder.
140. After 90 min using a clean scalpel or gel cutter per band, excise the band at ∼400 bp and split equally across 2 × 1.5 mL tubes.
141. Calculate the mass of the extracted gel slices.
142. Using the Qian QIAquick Gel Extraction kit, add 300 μL QG buffer per 100 mg of gel to each sample.
143. Incubate at 50°C for 10 min or until the gel slice has dissolved. Vortex every 2 min.
144. Once dissolved move to bench and add 10 μL 3 M Sodium Acetate and mix.
145. Into 2 mL lo-bind tubes add 100% isopropanol equal to the mass of the band extracted (i.e. for 100 mg add 100 μL isopropanol).
146. Transfer QG buffer containing melted gel and Sodium Acetate to the lo-bind tube containing isopropanol.
147. Mix by inversions.
148. Load 700 μL of sample onto a column.

***Note:*** You should have two tubes per band, if the sum-total weight of the two tubes is <400 mg you can combine the same samples onto one column. However if >400 mg continue to treat them separate.

149. Centrifuge at 18000 g for 1 min at room temperature.
150. Discard the flow through.
151. Repeat steps 148–150 until all QG buffer and isopropanol sample are added to their respective columns.
152. Add 750 μL wash buffer to each column and incubate at room temperature for 3 min.

**CRITICAL:** Don’t forget to add 100% ethanol to the wash buffer

153. Centrifuge at 18000 g for 1 min at room temperature.
154. Discard the flow through.
155. Centrifuge at 18000 g for 1 min at room temperature.
156. Transfer the column to a 1.5 mL lo-bind tube.
157. Add 30 μL EB directly to the membrane with a P200.
158. Wait 1 min.
159. Centrifuge at 18000 g for 1 min at room temperature.
160. Take eluate and add back to the column.
161. Wait 1 min.
162. Centrifuge at 18000 g for 1 min at room temperature.
163. Discard the column.
164. Combine eluate from the same sample into one and vortex.
165. Quantify the DNA quality using nanodrop and Qubit dsDNA High Sensitivity Assay.
166. Store the quantified DNA at −20°C.

### Pooling DNA for Illumina sequencing

**Timing: 1 day**

This section outlines the protocol for pooling each of the individual barcoded and indexed samples for next generation sequencing using Illumina.

**CRITICAL:** The concentration of the pooled library for sequencing will depend on the instrument used for sequencing and the service provider. You should sequence the library at a depth of at least 500× and ideally 1000× or more per gRNA in the library. Please check with your sequencing provider beforehand on how they would like the library prepared, including recommended PhiX concentration.

167. Use the Illumina pooling calculator to help calculate the library dilutions https://support.illumina.com/help/pooling-calculator/pooling-calculator.htm.
  a. Library Plexity = Number of samples to pool.
  b. Do the libraries have the same concentration? = Different (Qubit concentration).
  c. Unit of measure for the library = ng/μL (concentration from Qubit).
  d. Library Size = 365 (Size of PCR product).
  e. Pooled Concentration = Required Concentration by Sequencing Service.
  f. Total Pooled Library Volume = Required Volume by Sequencing Service.
168. Input the Library Concentration (ng/μL) for each sample as determined by Qubit.
169. Dilute the volume of cleaned PCR2 product provided in the “Library Volume (μL)” column in the volume of EB provided in “10 mM Tris-HCl, pH 8.5 (μL)” column.
170. Vortex to mix.
171. Into a fresh 1.5 mL lo-bind tube, combine the required volume of the diluted PCR2 products from each sample as outlined in the “Pooling Volume (μL)” column.
172. Vortex and submit for sequencing, using 150 bp paired end reads, with 5% PhiX control DNA.
173. Stored purified PCR2 products and diluted PCR2 products at −20°C.

### Data analysis and quality control

**Timing: 7 days**

This section of the protocol outlines the procedure for analyzing the gRNA abundances between the different populations using MAGeCK, a commonly used CRISPR screening analysis package. The section also outlines the recommended quality control checks and downstream packages used to assess the quality of the screen, as well as the quality of the hits.

**Table 12:** PCR2 cycling conditions for sequence indexing

Step	Temp	Time	Cycles
Initial Denaturation	98	30 sec	1
Denaturation	98	10 sec	10 cycles
Annealing	55	30 sec	
Extension	65	15 sec	
Final Extension	65	5 min	1
Hold	10	Forever

**Figure 7:**
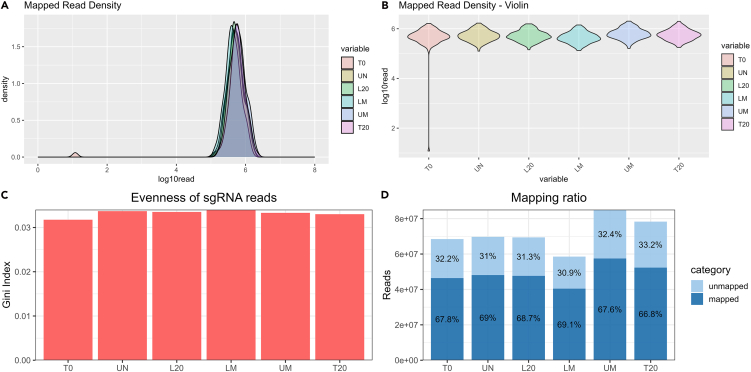
Quality control of MAGeCK mapping (A) Normal distribution of reads mapped to sgRNA indicates high coverage, bimodal distribution indicates very low coverage. (B) Violin plots showing the same data as (A). (C) Gini Indexes show if there is any drop out of sgRNA in the samples, all samples are <0.1 indicating good evenness of guide coverage. (D) Mapping rates of the sequencing reads to sgRNA sequences, all samples are mapped very highly (>65% across all samples), this can vary dependent on the sequencer used, using the forward or reverse read, or using the complement or reverse complement sgRNA sequencing in the MAGeCK Count steps.

**Figure 8:**
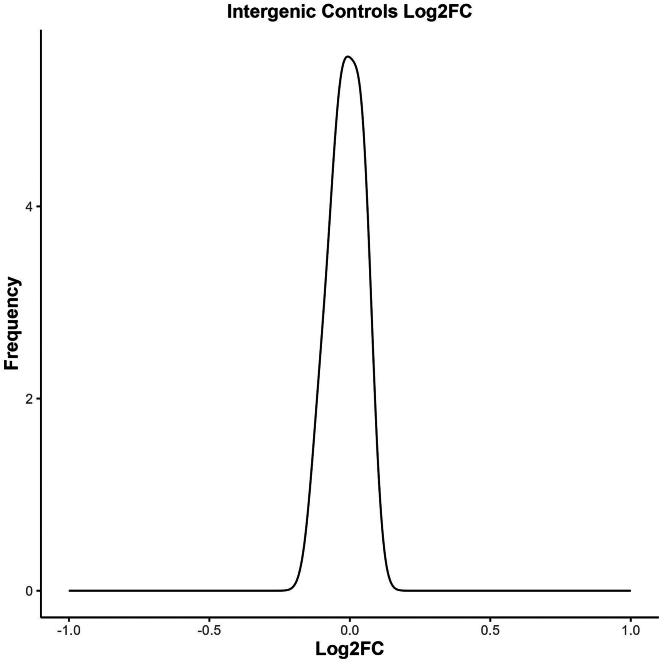
Normal distribution of the Log2FC for the control sgRNA This is expected as we normalized the results to the Intergenic Controls. There should be no observed enrichment with the control genes. If there is a shift, this indicates that the normalization has not worked. If normalizing to the library size you would expect your control sgRNA to have no effect, if there is a strong effect then any resultant hits could be due to noise in the assay.

**Figure 9:**
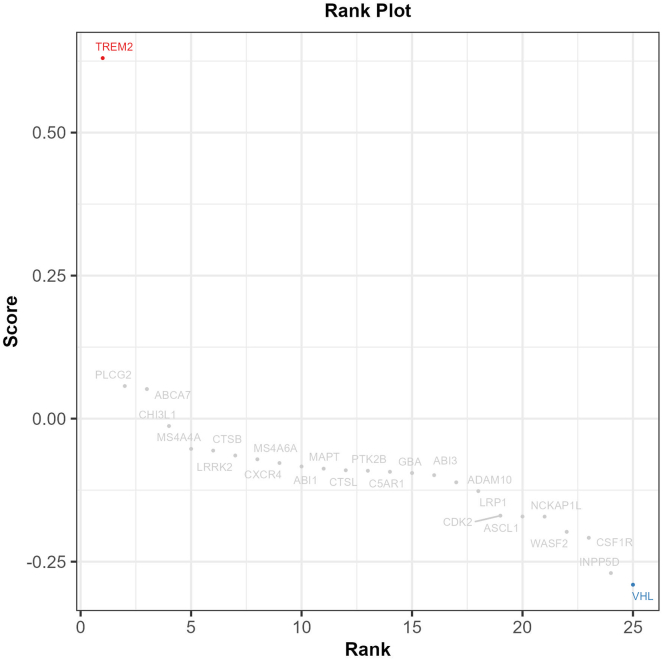
An example rank plot for neuronal phagocytosis in iMGL shows *TREM2* knockout increases autophagy, whereas *VHL* knockout decreases phagocytosis Other known control genes are moving in a similar direction, such as *WASF2, NCKAP1L.* This can be a good extra quality control, to have strong positive and negative controls.

**Figure 10:**
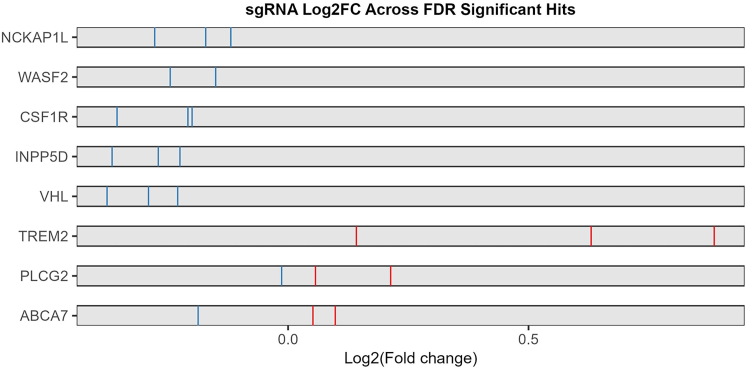
sgRankView of the FDR significant hits from the example screen This shows the individual gRNA Log2FC between the tested and control populations.

**Figure 11:**
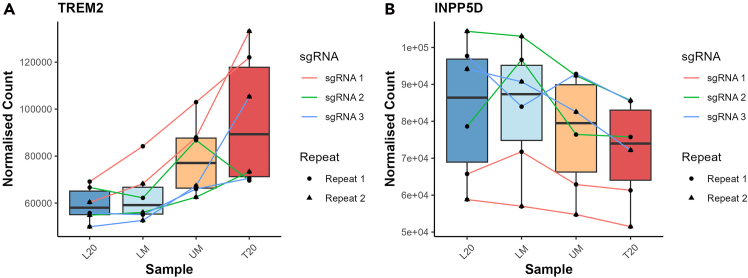
Example plots showing the sgRNA abundances across the four sorted populations can provide additional confirmation that the genetic knockout is not down to sporadic changes in population (A) TREM2 shows an increase in sgRNA abundance as the phagocytosis increases, this means that TREM2 KO increases phagocytosis. (B) INPP5D shows the opposite, a decrease in sgRNA abundance as the phagocytosis increases, this means that INPP5D KO decreases phagocytosis.

There are several components to the screen analysis, of which these can be split into the following:

 I. QC of raw sequencing reads – MultiQC – Bash.

 II. Merging of multiple fastq files of the same samples – Samtools – Bash.

 III. Counting sequencing reads mapping to sgRNA library – MAGeCK – Bash.

 IV. Differential sgRNA abundances – MAGeCK – Bash.

 V. Downstream analysis – MAGeCKFlute - R.

**CRITICAL:** The analysis should be carried out by someone with competency in Unix/Linix, Bash, R. While the code provided can be replicated the underlying understanding of the analysis should be known. Most analysis can be carried out on a standard MacOS or Windows system (with Ubuntu installed).

174. Download the .fastq files from the sequencing service.

**CRITICAL:** Backup the files in several locations as they are not usually stored long term at sequencing service providers.

175. Undertake a first round QC using MultiQC – checking for high Phred Scores (>30). There will be a high number of duplicate sequences due to the nature of sequencing a PCR library.
176. Install MAGeCK on your machine using bioconda: https://anaconda.org/bioconda/mageck.
177. Prepare the library metadata file for MAGeCK.^12^ The formatting is very specific and has to contain the following headers and column order: GUIDE_ID, SEQUENCE, GENE. Where GUIDE_ID is the chr location, gene name and strand (chr19:58864777-58864796_A1BG_+), SEQUENCE is the guide sequence excluding PAM, and GENE is the gene name/identifier, this can be Ensembl or Symbol.
178. It is also recommended to run multiple quantifications using both the Forward and Reverse reads with the Forward and Reverse Complement sgRNA sequences to achieve maximal mapping for MAGeCK Test. You would expect to see between 60%–80% mapping efficiency. See the (Table 12) for the suggested quantifications:
179. Run the quantification of the sgRNA abundances using MAGeCK count.

>Mageck count –l library.csv –fastq sample1.fastq.gz sample2.fastq.gz sampleN.fastq.gz…. –sample-label Sample1, Sample2, SampleN… -n outputfilename

The main arguments in the above code are:

 -l: this is the sgRNA library used (see step 177).

 -fastq: this is a list of the FASTQ files separated by a space. Note, only include the forward or reverse fastq files not both.

 -sample-label: this is the column labels for the read count matrix. These should match the order of the input FASTQ files. If the incorrect label order is provided the output read table will be incorrect.

 -n: this is the output file name.

180. Once the analysis is complete (could take several hours) there will be several files. The important files are the countsummary.txt which provides mapping information, the .log file which provides any errors, and the .count.txt which is the count matrix for downstream analysis.
181. Run the QC of the mapping using R and the package MAGeCKFlute^13^ (Figure 7).
  a. Density plots of the read counts – Check the distribution of the reads mapping each sgRNA, there should be a clean single peak normally distributed.
    **CRITICAL:** If there are multiple peaks or a bi-modal distribution STOP, the coverage of the library is likely too low to give meaningful data and the guide distributions are likely to be random.
  b. Mapping ratio – this is the data from the countsummary.txt which includes what percentage of the sequence reads are mapped to a known sgRNA per sample. This can tell us if the sequencing has been successful. For reference, samples should be >60% mapped. This can vary between sample or between the read/library orientation in the MAGeCK count.
  c. Gini Index – this is used to calculate sgRNA drop out across the samples. A lower Gini index indicates fewer drop outs. A good Gini Index is considered <0.1.
182. The next step is to quantify the sgRNA abundances across the populations to act as a proxy for genetic knockout. Select the count file which gives the highest quality (normal distribution of mapped reads, highest mapping %, lowest gini indexes).
183. There are several comparisons you can do with the data;
  a. Drop out screening – Using the T0 as the reference and unsorted populations as the test you can determine if any genes result in toxicity through the iMGL differentiation from iMGL precursor to iMGL.
    ***Note:*** This can be used as an additional QC measure Cas9 activity particularly if the lentiviral library contains known essential genes. This can validate that editing is occurring.
  b. FACS-Paired Screening (L20 vs T20) – Using the L20 as the reference and the T20 as the test you can determine if any genes result in an increase in your phenotype of interest. This is the most common comparison.
  c. Comparing 3 or more populations - This can be achieved through the MLE model, for example a four way sort on two populations to give high high, high low, low high, and low low. This is a more complex analysis and is not examined in this protocol.
184. To perform the differential sgRNA analysis run the following code to run the MAGeCK RRA:

>mageck test –k count_table.txt –t colname_test_screen1,colname_test_screen2,colname_test_screen3 –c colname_cont_screen1,colname_cont_screen2,colname_test_screen3 –n outputfilename –normcounts-to-file

The main arguments in the above code are:

 -k: this is the count table generated from MAGeCK count.

 -t: these are the column names for the test variables, either UN or T20.

 -c: these are the column names for the control variables, either T0 or B20.

 -norm-method: this sets the normalization method to the controls within the library, rather than normalizing by the library size. Normalizing to controls provides increased power to detected changes if you know the controls should not influence the measured phenotype.

 -control-sgrna: this is a list of the control sgRNA names for the –norm-method if set to control, this needs to match to the sgRNA list in the count table row names.

 -n: this is the output file name.

 -normcounts-to-file: this outputs the normalized read counts to a .csv file which is useful for visualization in MAGeCKFlute.

***Note:*** You can also normalize to library size, this is the default setting within MAGeCK test, this assumes that all of the genes in the library are non-essential however the significance associated will be over-conservative and there will be a lot of false-negatives. It is down to the discretion of the user to decide the best normalization method for their data.

185. Once the analysis is complete there will be several files. The important files are the .sgrna_summary and the .gene_summary which provides the output of the significant sgRNA and genes respectively. The .log file will provide any errors, and the .normalized.txt which is the normalized count matrix for downstream analysis in R.
186. Check distribution of the control genes to confirm normalization has worked. Within the library there should be a set of control genes, of which you would expect these to have no effect on your assay. For example, the TKOv3 contains sgRNA targeting EGFP, Luciferase, and LacZ which should not be present in your cell line. The library used for this work contained 8 intergenic sgRNA which cut at intergenic sites, plot the Log2FC from the .sgrna_summary. These should give a normal distribution around Log2FC 0 to confirm the normalization has worked (Figure 8).

***Note:*** If using library size as normalization it is good to check the distribution of control sgRNA within the library. These should be near 0 Log2FC, any enrichment would indicate noise within the screening phenotype as you would expect these controls to have no effect.

187. We have confirmed good coverage of the library and have confirmed that the control guides are having no effect on the phenotype, therefore we can now examine the individual genes which, when knocked out, result in a phenotype, this is through ranking the Log2FC and the FDR changes and plotting these as a Rank Plot using the RankView function. Anything in Red, knockout results in an increase of the observed phenotype, anything in Blue, knockout results in a decrease of the observed phenotype. In our example of phagocytosis, TREM2 is red, indicating knockout increases phagocytosis, whereas VHL is blue, indicating knockout decreases phagocytosis (Figure 9).
188. Next you can pull out any FDR corrected hits, as the rank plot does not give you this, it only gives you a visual representation.
189. As MAGeCK RRA collapses the sgRNA abundance into gene level scores, it is also worth exploring the individual gRNA Log2FC of significant hits. This can be done with the sgRankView function (Figure 10).
190. Finally, because we have data across four sorted pools we can plot the normalized count data of the individual gRNA across the repeats. This can further confirm that the gRNA abundances are changing across the groups and can further validate that the targets are not down to sporadic changes or sampling biases (Figure 11).
191. A list of hits for further validation can then be selected by the user, we recommend using a combination such as:
  a. Log2 Fold Change at the gene level.
  b. Consistency of the direction of effect in the individual sgRNA.
  c. Adjusted P Value.
  d. Are there multiple hits within the same pathway.

### Validation of screening hits

**Timing: 6–12 months**

This section of the protocol outlines the recommended further validation of the CRISPR screening hits which should be used to confirm the validity of the findings.

Dependent on the size of the library used for screening you may end up with hundreds of possible hits. It is therefore important to validate any findings to confirm these are robust. This could be done in several ways and are down to the discretion of the scientist.

192. Performing the same phenotypic assay in an arrayed CRISPR screening format, where individual genetic knockout is per well.
193. A different phenotypic assay which measures a similar readout, as the assay presented here is a FACSs based the assay could be repeated using imaging based readouts.
194. Utilizing a different method of perturbation such as Cas9 ribonucleoprotein, RNA inhibition, antisense oligonucleotides, or pharmacological inhibition, and measuring the same phenotypic readout.
195. In experiments utilizing hiPSC it is **highly recommended** to do any validation in multiple genetic backgrounds (including the background used for the screen itself), to confirm findings are not a result of the background genetics of the KOLF2.1J line. We recommend another line from the HipSci collection to account for differences in iPSC generation, we also recommend a female line to account for sex differences.

## Expected outcomes

As an example of expected outcomes, see the original article (Perez-Alcantara 2025). In the online GitHub repository it is possible to see the raw and normalized count files as well as the outcome hits from the downstream analysis using MAGeCK and potential validations strategies to confirm the validity of the screen.

## Limitations

There are several limitations of the described protocol. A major limiting factor is maintaining the coverage throughout the screen and sorting enough cells to provide high enough coverage for meaningful data. This can be exceptionally difficult when using large genome-wide CRISPR libraries, therefore it is vital to assess if it will be feasible to grow and maintain enough iMGL for the required coverage at the end of the screen. Secondly, this protocol requires a large number of tissue culture incubators for the growth and maintenance of the factories, before commencing it is crucial that there is enough space within the laboratory for extended culturing of the iMGL. Finally, this protocol can be easily adapted to different hiPSC cell lines, however changing the line can have a big impact on the number of iMGL precursors and the timing of their release into the supernatant of the factory media. It is vital therefore to optimize the initial differentiation using the cell line of choice if not using the KOLF2.1J background.

## Troubleshooting

### Problem 1

hiPSC do not form embryoid bodies. Related to step 8.

### Potential solution

• Check concentrations and dilutions of cytokines and growth factors for EB media.
• Check genetic integrity of the hiPSC through SNP array or whole genome sequencing to confirm no chromosomal alterations have been introduced during passaging.
• Increase the frequency of EB media changes.
• Work faster when preparing the cell suspensions, delays in this step can reduce viability of the hiPSC.

### Problem 2

Factories do not produce precursor cells. Related to step 12–17.

### Potential solution

• Check concentrations and dilutions of cytokines and growth factors for factory media.
• Are the EB attached to the flasks and have formed stromal skirts?, if they have not repeat EB generation from Step 8.

### Problem 3

Factories produce low quality precursors cells with low viability, Related to step 12–17.

### Potential solution

• Are these early harvests? D5-D15 usually contain lots of dead/dying cells as the precursors have not yet been produced to clear the debris.
• Perform larger media changes on the factories to remove apoptotic cells, up to 80% of the factory media can be replaced.
• The media could have been exhausted, increase the frequency and volume of feeds.

### Problem 4

Inefficient production of VPX-VLPs or lentiviral library. Related to steps 38–52.

### Potential solution

• Plasmid DNA must be of high quality and concentration, check on an agarose gel for DNA integrity.
• Check for mycoplasma contamination within the HEK293T.
• HEK293T cells must be passaged at least twice prior to transfection and no more than 20 times.
• Do not overgrow the HEK293T cells during expansion.
• When performing media changes at 24 and 48 h post transfection try to reduce the number of HEK293T removed from the plates by pipetting slowly. Removal of HEK293T cells can reduce the titer.

### Problem 5

Low transduction efficiency of the precursors. Related to step 52.

### Potential solution

• Potential loss of lentiviral titer during storage through repeated freeze thaw cycles, ensure storage of single use aliquots of the virus.
• The age of the precursors can influence transduction efficiency. In our hands using the same lentiviral prep older precursors are harder to transduce due to a lower number of cycling cells.
• Poor lentiviral titer during production.
• Not enough VPX-VLPs as determined by western blot, increase the concentrations of VPX-VLPs, or did you forget to add the VPX-VLPs during initial transduction.
• Scaling of the transduction from the titration experiment from 96 well plate to T175 is not 1:1, repeat titrations in the format in which the experiments will be undertaken.
• Check the seeding density and media volume.

### Problem 6

Low cell viability after transduction. Related to Step 61.

### Potential solution

• Lentiviral toxicity, reduce the concentration of the lentiviral construct.
• Reduce time taken to produce the transduction mixes and plating of the cells.

### Problem 7

Low DNA concentration or quality following extraction. Related to step 109.

### Potential solution

• DNA might still be present in WA or WB tubes, adjust to 0.3 M Sodium Acetate and repeat the isopropanol precipitation step.
• DNA might have been aspirated during the wash steps, ensure to use glycogen or glycoblue to visualize the pellet to prevent accidental aspiration.
• Residual ethanol carry over from improper drying, repeat the precipitation as described above and ensure to dry the DNA pellet until it has just become opaque.

### Problem 8

No bands or incorrect bands present during PCR reactions. Related to steps 118 and 132.

### Potential solution

• Check you are using the correct primer sequences for the vector being used for the lentiviral library. We have provided two primer sets for use with the all-in-one CRISPR v3 single guide backbone, and the all-in-one CRISPR v3 dual guide backbone.
• Incorrect number of cycles, for PCR 1 the number of cycles may be too low if the input DNA is very low of the number of untransduced cells are very high. Increase the cycle count and take 2 μL of PCR product every 2 cycles and run on an agarose gel. Do not go above 30 cycles as this can introduce biases into the library.
• Check the quality of the input DNA, if the volume is very high (i.e >10% of the total reaction) there could be residual salt carry over which would inhibit the PCR reaction.

### Problem 9

Uneven library distribution following sequencing. Related to step 174.

### Potential solution

Check dilutions of PCR 2 in Steps 167–172 and re-sequence if the library in very uneven.

### Problem 10

Issues assigning sequencing reads to library sequences. Related to step 179–181.

**Table 13:** Recommended combinations for sgRNA counting

Name	Sequence read	gRNA library
Forward_Comp	Forward (R1)	Complementary
Forward_RevComp	Forward (R1)	Reverse Complement
Reverse_Comp	Reverse (R2)	Complementary
Reverse_RevComp	Reverse (R2)	Reverse Complement

### Potential solution

• Check the formatting of the library.csv. MAGeCK has very specific requirements for the input of the library for mapping.
• Check that you are using the correct strandedness of the gRNA library for the sequencing read. Related to Table 13.
• Check sequencing quality through MultiQC as outlined in step 175.

### Problem 11

Uneven density of the sequencing reads, high Gini index. Related to Step 181.

### Potential solution

• Most likely due to low coverage of the library at one or more steps of the protocol. If you have spare DNA repeat the PCR 1 and re-sequence.
• If the above results in the same findings you will have to repeat the screen from step 53 increasing the number of cells to maintain the required coverage.
• Sequence the initial plasmid library or lentiviral prep to confirm the presence of the library and the distribution of the sgRNA, if there was low coverage to begin with this would have been amplified during the screen.

## Resource availability

### Lead contact

Requests for further information and resources should be directed to and will be fulfilled by the lead contact, Dr. Sam Washer, sam.washer@cmd.ox.ac.uk.

### Technical contact

Technical questions on executing this protocol should be directed to and will be answered by the technical contact, Dr. Sam Washer, sam.washer@cmd.ox.ac.uk.

### Materials availability

• Plasmids have been deposited to Addgene as described in the key resources table.
• Cell Lines were purchased from the Jax catalog or from ATCC as described in the key resources table.

### Data and code availability

• The datasets and code have been published and are available at https://github.com/S-Washer/Analysis_of_FACS_based_CRISPR_screens_in_iMGL
• The archived analysis can be found at https://doi.org/10.5281/zenodo.15862104

## Acknowledgments

This work was supported by a research grant from Open Targets (OTAR2065) awarded to S.A.C., D.V.E., and A.R.B. The bioreactor modules were supported by a small equipment grant from Alzheimer’s Research UK Thames Valley Network awarded to S.J.W.

## Author contributions

S.J.W. performed all laboratory experiments and analyzed the data. S.J.W. and E.N.-G. prepared the manuscript. S.J.W., E.N.-G., S.A.C., D.V.E., and A.R.B. discussed results and contributed to the final article.

## Declaration of interests

A.R.B. is a founder and consultant for Ensocell Therapeutics.

## Supplemental information

Document S1. Figure S1

Table S1. Cell number calculation sheet, related to step 53

Table S2. Primer sequences for next generation sequencing library preparation, related to step 111
