# Supplementary material for: Protocol for pooled FACS-based CRISPR knockout screening in human iPSC-derived microglia
Source: STAR Protoc. 2025 Sep 20;6(4):104111. doi: 10.1016/j.xpro.2025.104111 (PMC12483672; doi:10.1016/j.xpro.2025.104111)
Supplement: Document S1. Figure S1 [file mmc1.pdf]

Supplementary Files:

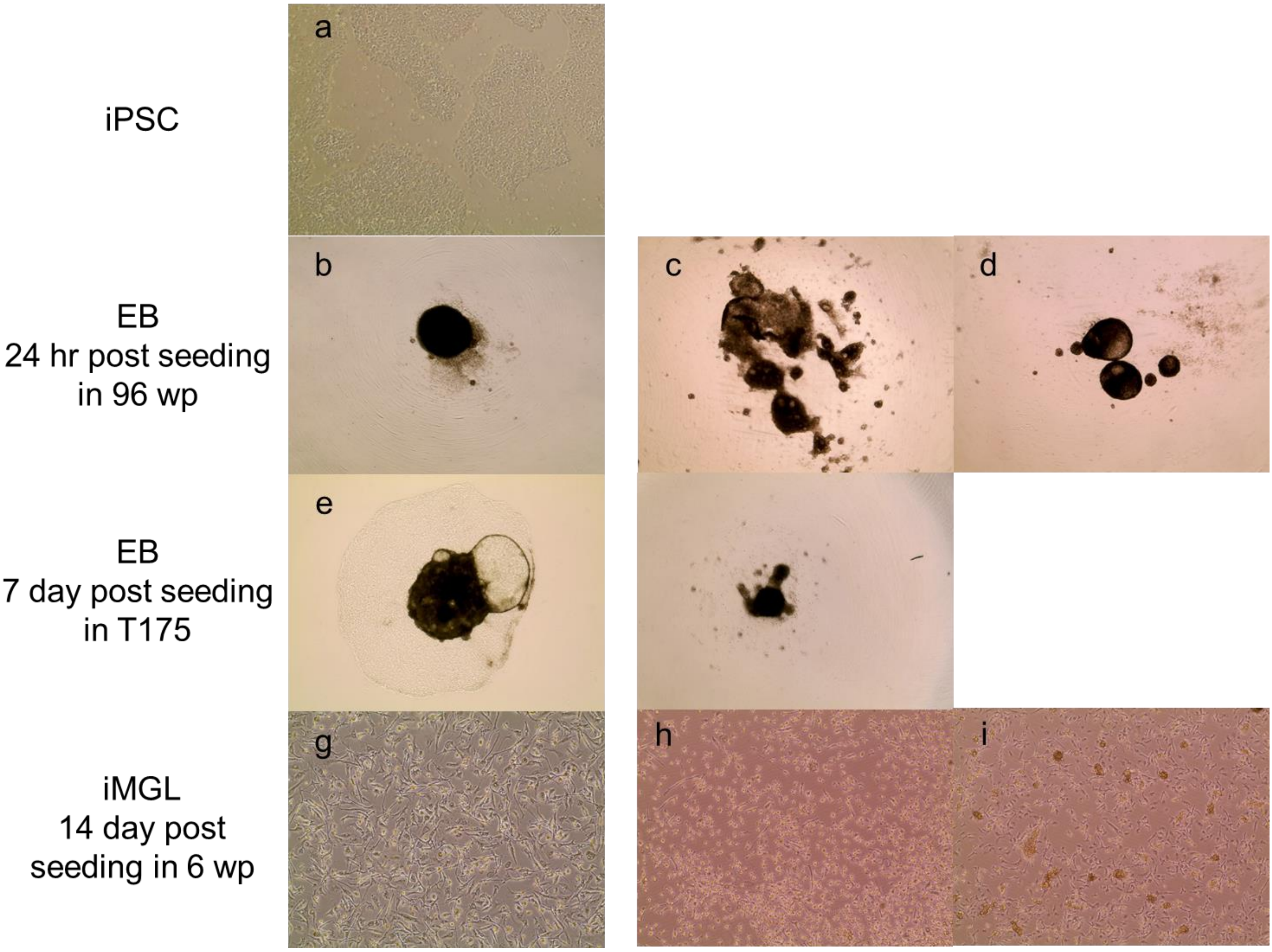
iPSC
a
EB
24 hr post seeding
in 96 wp
b
c
d
EB
7 day post seeding
in T175
e
iMGL
14 day post
seeding in 6 wp
g
h
i

Figure S1: Example images of the iMGL differentiation, related to step 7: A) Example iPSC confluence required for seeding the embryoid bodies (EB). B) A well-defined and formed EB 24 hours post-seeding in a 96 wp U bottom low attachment plate. C and D) Failed EB generation at 24 hours post-seeding. C) has failed to form a defined EB whereas D) has produced multiple “mini” EB in a single well. E) An Example EB 7 days post-seeding into a T175 factory, showing a well defined and clear stromal skirt, the EB is in the centre, with an air sac shown on the right. F) A failed EB within a factory 7 days post-seeding, shows no defined edges, no stromal skirt, reduced size, and disintegration of the 3D structure. G) Example iMGL 14 days post-seeding in ITMG, showing even coverage of the surface with few detached cells. H) An example of loose iMGL at day 14 post-seeding, showing a more rounded morphology, with more bare space and a lower confluency. I) An example of stressed iMGL showing cellular clumps. Both H and I are examples of a poor iMGL differentiation from the precursor cells.
